# Occlusal types shape oral microbiome stomatotypes and metabolic landscapes: A multi-omics perspective on host-microbe interaction

**DOI:** 10.15698/mic2026.06.879

**Published:** 2026-06-15

**Authors:** Yufeng Duan, Zheng Liu, Wenting Lu, Ning Zhao, Lingjun Yuan, Zhenxia Li, Ting Zhou, Shengzhao Xiao, Dian Jing, Xiaowen Zheng, Wentao Shi, Chao Liu, Haixia Lu, Qiang Feng, Bing Fang

**Affiliations:** 1 Department of Orthodontics, Shanghai Ninth People’s Hospital, College of Stomatology, Shanghai Jiao Tong University School of Medicine; National Center for Stomatology; National Clinical Research Center for Oral Diseases; Shanghai Key Laboratory of Stomatology, Shanghai 200125, China.; 2 Center for Reproductive Medicine, Shandong University, Jinan, Shandong, 250012, China.; 3 Senior Department of Obstetrics and Gynecology, Chinese PLA General Hospital, Beijing 100853, China.; 4 Department of Clinical Research Center, Shanghai Ninth People’s Hospital, College of Stomatology, Shanghai Jiao Tong University School of Medicine, Shanghai 200125, China.; 5 Department of Preventive Dentistry, Shanghai Ninth People’s Hospital, College of Stomatology, Shanghai Jiao Tong University School of Medicine, National Center for Stomatology; National Clinical Research Center for Oral Diseases; Shanghai Key Laboratory of Stomatology, Shanghai 200125, China.; 6 Department of Human Microbiome, School and Hospital of Stomatology, Cheeloo College of Medicine; Shandong Provincial Key Laboratory of Oral Tissue Regeneration; Shandong Engineering Laboratory for Dental Materials and Oral Tissue Regeneration Shandong University, Jinan 250012, Shandong, China.

**Keywords:** occlusal type, oral microbiome, metabolomics, stomatotypes

## Abstract

Clinical studies have uncovered associations between malocclusions and bacteria-related oral diseases. However, which malocclusion drives alternations in the oral microbiome remains unclear. Here, we identified occlusal type (OT, a major malocclusion classification parameter) as a key host structural regulator of the oral microbiome composition and metabolite profiles in adolescents. Regarding microbial composition: *Prevotella* and *Veillonella* species enriched in the OT-I group, whereas *Neisseria* and *Haemophilus* species predominated in the OT-II group. These differential distributions and unique microbial associations contributed to the formation of two distinct oral microbiome clusters (“stomatotypes”). In terms of gene functions, the OT-II group exhibited enrichment in “Environmental information processing” (EIP) pathways, “Human Diseases” (HD) pathways, and virulence-associated genes including relA and cpsB/cdsA. Significant differences in metabolite profiles were also observed between groups. Multi-omics analysis revealed positive intra-group associations and negative associations between-groups among representative oral microbes, functional pathways, and metabolites, with specific dipeptides identified as potential key microbe-modulated metabolites. Our results revealed the pivotal role of OT in shaping the variations of the oral microbiome and metabolite, offering novel insights into how host anatomical structure influences oral microecology.

## INTRODUCTION

The oral microbiome dysbiosis not only serves as a key driver of various oral diseases 1 but also is associated with systemic implications, including type 2 diabetes and inflammatory bowel disease 2, 3. Emerging evidence suggests that the oral microbiome dysbiosis is more like a dynamic and polymicrobial change in species composition and function profiles, influenced by both host physiology and external environmental factors 4, 5. External stimuli, like cigarettes and alcohol, have been confirmed as long-term pathogenic promoting factors that interfere with the oral microbiome, leading to systematic alterations in microbial composition and function 5–7. For example, clinically healthy e-cigarette users show pathogen overexpression, higher virulence profiles, and active pro-inflammatory signaling in their oral microbiome, similar to patients with severe periodontitis 7. However, the role of intrinsic anatomical variations, particularly malocclusion, in shaping the oral microbiome remains poorly understood.

Malocclusion, affecting 48–81% of the global population, ranks as the third most prevalent oral health issue 8. Clinical studies correlate several malocclusion subtypes with bacterial-related oral diseases. For example, malocclusion correlates with increased risk of dental caries in adolescents and children 9, 10, and distal occlusion shows positive associations with dental caries in adults 11, as well as the dental plaque and periodontal disease 10, 12, 13. Mechanistically, malocclusion-related structural anomalies may contribute to these oral diseases by altering the physical microenvironment of the oral cavity. For instance, crowding may disrupt the natural physical self-cleaning and salivary flushing mechanisms, leading to localized nutrient and dental plaque retention 14. This microenvironment shift, in turn, may exerts selective pressure on the colonization and growth of specific microbial communities.

As the occlusal type (OT) can obviously affect the anatomy of the oral cavity, we hypothesize that OT can modulate oral microbiome composition and function. However, the relationship between OT and oral microbial dynamics remains unknown. Therefore, by performing integrated metagenomic and metabolomic profiling on saliva samples from adolescents with OT-I and OT-II, we sought to characterize the distinct patterns of oral microbiome composition, functional pathways, and metabolic profiles associated with OTs.

## RESULTS

### Basic characteristics

**Figure 1:**
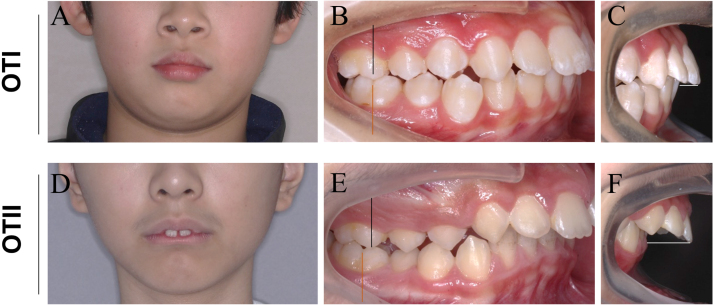
Facial and intraoral photograph of representative occlusal type (OT) I and II. **(A)** Facial view of OT-I: Showing natural lip closure with relaxed upper and lower lips. **(B)** Intraoral view of OT-I: The buccal cusp of the maxillary first molar (marked by black short lines) aligns with the buccal groove of the mandibular first molar (marked by brown short lines). **(C)** Intraoral view of OT-I: Overjet size is illustrated by a white horizontal line. **(D)** Facial view of OT-II: Incomplete lip closure with tension in the upper and lower lips, accompanied by anterior tooth exposure. **(E)** Intraoral view of OT-II: The buccal cusp of the maxillary first molar (black short lines) is positioned mesial to the buccal groove of the mandibular first molar (brown short lines). **(F)** Intraoral view of OT-II: A relatively larger overjet compared to OT-I.

This study enrolled 54 healthy adolescents (30 OT-I, 24 OT-II) with a mean age of 11.6 years, and no significant intergroup differences in age, sex distribution (all *P* > 0.05; Table 1). The representative facial and intraoral pictures of OTI and OTII were presented in the Figure 1. Both groups exhibited comparable height (OT-I: 154.48 ± 6.70 cm vs. OT-II: 156.29 ± 7.14 cm), weight (46.12 ± 10.69 kg vs. 46.41 ± 8.53 kg), and BMI (19.22 ± 3.84 vs. 18.91 ± 2.72 kg/m ${}^{2}$ ). Oral health assessments revealed similar DMFT scores (OT-I: 0.19 ± 0.40 vs. OT-II: 0.25 ± 0.44) and dental crowding patterns (upper arch: 1.69 ± 2.07 mm vs. 2.25 ± 2.10 mm; lower arch: 1.25 ± 1.13 mm vs. 1.45 ± 1.58 mm), suggesting comparable baseline caries risk and arch crowding characteristics.

Notably, the OT-II group presented significantly greater overjet compared to OT-I (5.15 ± 2.70 mm vs. 3.50 ± 1.13 mm, P = 0.009), consistent with Class II malocclusion diagnostic criteria. Overbite also trended higher in OT-II (4.42 ± 1.69 mm vs. 3.71 ± 1.17 mm, P = 0.096, Table 1). These results confirmed the successful classification of participants by occlusal type while controlling for major demographic and anthropometric confounders. Their detailed characteristics are presented in Table 1, Supplemental Table 1–3.

**Table 1:** Characteristics of the cohort in OTI and OTII.

	**OTI(N = 30)**	**OTII(N = 24)**	***P* value **
**Age (Y)**	11.58 ± 0.31	11.64 ± 0.27	0.49
**Sex**	Female: Male = 18:12	Female: Male = 15:9	0.729
**Height (cm)**	154.48 ± 6.70	156.29 ± 7.14	0.365
**Weight (kg)**	46.12 ± 10.69	46.41 ± 8.53	0.917
**BMI (kg/m** ${}^{2}$ **)**	19.22 ± 3.84	18.91 ± 2.72	0.743
**DMFT**	0.19 ± 0.40	0.25 ± 0.44	0.631
**Crowding at upper teeth (mm)**	1.69 ± 2.07	2.25 ± 2.10	0.350
**Crowding at lower teeth (mm)**	1.25 ± 1.13	1.45 ± 1.58	0.593
**Overjet (mm)**	3.50 ± 1.13	5.15 ± 2.70	0.009(**)
**Overbite (mm)**	3.71 ± 1.17	4.42 ± 1.69	0.096

Data were presented with mean ± SD, For the difference comparison of clinical characteristics between the groups, student’s t-test (two-tailed) was applied for continuous data. Categorical variables were compared by the χ 2 test.

**Figure 2:**
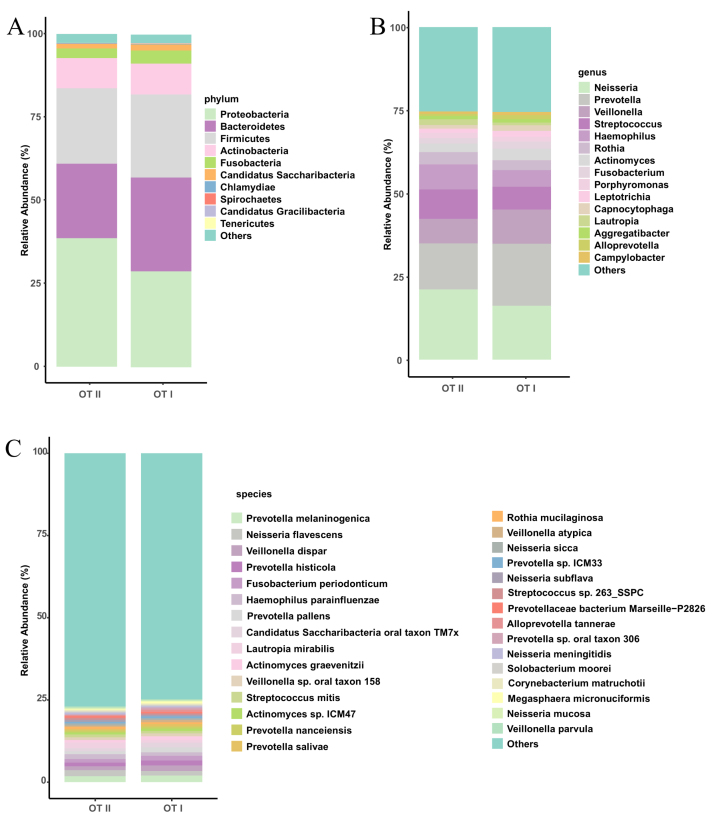
The oral microbiome profile of the OT-I and OT-II groups. The microbiome composition of the OT-I and OT-II groups at the phylum **(A)**, genus **(B)** and species **(C)** level were presented by the relative abundance.

Metagenomic sequencing generated about 6GB of high-quality data per sample, with bacterial sequences constituting 90% of total annotations, while viral components representing only 0.2% (Fig. S1A). Rarefaction curves confirmed sufficient sequencing depth for capturing microbial diversity at both taxonomic and functional levels (Fig. S1B, S1C). The oral microbiomes of OT-I and OT-II groups exhibited a conserved phylum-level structure, with *Proteobacteria*, *Bacteroidetes*, *Firmicutes*, *Actinobacteria* and *Fusobacteria* collectively representing over 90% of the bacterial communities (Figure 2**A**, Fig. S2), which is consistent with previous reports 15. At the genus level, *Neisseria*, *Prevotella*, *Veillonella*, *Streptococcus*, *Haemophilus* constituted the dominant taxa (relative abundance > 50%; Figure 2**B**), with *Prevotella spp*. (*e.g.*, *P. melaninogenica*) and *Neisseria spp.* (*e.g.*, *N. flavescens*) emerging as the most abundant species (Figure 2**C**). Additionally, Complementary metabolomic profiling via UPLC-MS detected 491 metabolites, including microbiome-associated dipeptides (Supplemental Table 4), enabling subsequent multi-omics integration.

### The occlusal type significantly influenced the composition and diversity of the oral microbiome

Four methods-Adonis (R ${}^{2}$ = 0.035, P = 0.036), dbRDA (F = 1.853, P = 0.031), MRPP (A = 0.008, P = 0.019), and Anosim (R = 0.051, P = 0.046)-consistently revealed significant microbial divergence between different occlusal types (Table 2).

**Figure 3:**
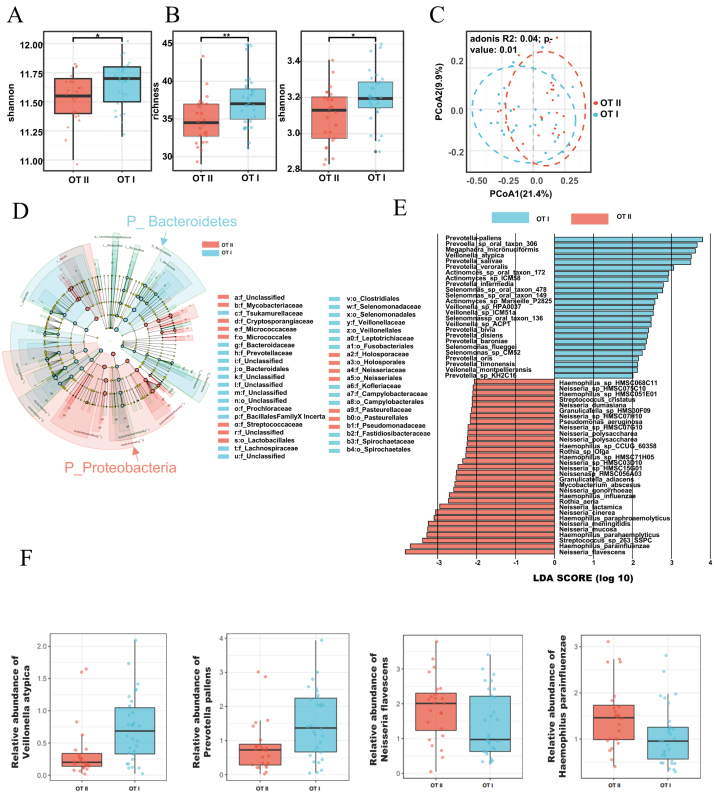
Differences in Oral Microbiome Between OT-I and OT-II Groups. **(A)** The oral microbiome in the OT-II group showed obviously lower α diversity at both the gene level (Shannon index), and **(B)** species level (richness and Shannon index) compared to the OT-I group, indicating a more uniform distribution of genes and species in the OT-I groups. **(C)** PCoA revealed distinct β -diversity between the OT-II and OT-I groups. **(D)** A cladogram generated by LEfSe highlights differentially abundant taxa, with blue nodes indicating enrichment in OT-I and red nodes indicating enrichment in OT-II. **(E)** Differentially abundant species between the two groups are shown, ordered by LDA scores, with colors corresponding to the respective sample types. **(F)***Prevotella pallens* and *Veillonella atypica* are the most representative species in the OT-I group, while *Neisseria flavescens* and *Haemophilus parainfluenzae* are most representative in the OT-II group.

The OT-II group showed reduced alpha diversity compared to OT-I, with lower Shannon indices and species richness (Figure 3**A**, 3**B**). Beta diversity analysis with PCoA further confirmed distinct community structures between groups (Adonis, P < 0.05; Figure 3**C**).

**Table 2:** Potential parameters which affect the oral microbiome were examined by PERMANOVA.

	**Adonis**		**dbRDA**		**MRPP**		**Anosim**	
**Indicators**	**R2**	**p-value**	**F value**	**p-value**	**A value**	**p-value**	**R value**	**p-value**
**Occlusal type (OTI, II)**	0.035	0.018	1.859	0.028	0.008	0.019	0.051	0.046

LEfSe analysis identified OT-associated microbial biomarkers: The major clades of these biomarkers were plotted on the cladogram (Figure 3**D**), and key species were ranked by their Linear Discriminant Analysis (LDA) score (Figure 3**E**). Notably, *Prevotella* and *Veillonella* species *(e.g., P. pallens and V. atypica) were significantly* enriched in OT-I*,* whereas *Neisseria* and *Haemophilus* species *(e.g., N. flavescens; H. parainfluenzae)* enriched in OT-II. (Figure 3**E**, 3**F**).

### The occlusal type affects the oral microbiome stomatotypes

**Figure 4:**
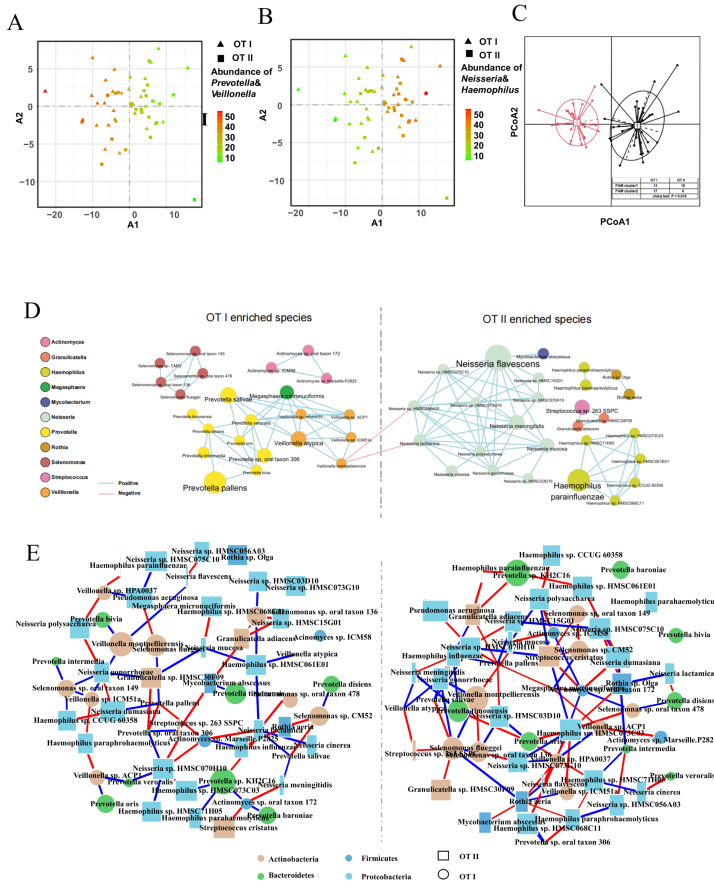
Group-specific interactions of differential oral microbiome in OT-I and OT-II groups. **(A)** The sum of abundances of the “*Prevotella*-*Veillonella*” is significantly higher in the OT-I samples. **(B)** The sum of abundances of the “*Neisseria*-*Haemophilus*” is significantly higher in the OT-II samples. **(C)** PCoA analysis showed distinct clustering of samples into two groups, with a significant difference in the distribution of OT-I and OT-II samples across PAM clusters 1 and 2. **(D)** Co-occurrence networks of differential species in OT-I and OT-II group revealed three major clusters, with blue lines indicating positive correlations and red lines indicating negative correlations. Node size reflected mean species abundance. **(E)** SparCC networks of differential species in groups OT-I and OT-II were constructed. The red and blue connecting lines represented positive and negative correlations, respectively. The node size represented mean species abundance.

Our LEfSe analysis identified species of “*Prevotella*-*Veillonella*” and “*Neisseria*-*Haemophilus*” as key biomarkers of OT-I and OT-II groups (Figure 3**E**, Fig. S3). Importantly, samples from the OT-I groups were largely classified into the ”*Prevotella-Veillonella*” stomatotypes, while those from the OT-II group largely clustered into the ”*Neisseria-Haemophilus*” stomatotypes (Figure 4**A**, 4**B**). Suggesting the occlusal type may affect the oral microbiome stomatotypes.

To further investigate the association between the occlusal type and oral microbiome stomatotypes, we applied the Dirichlet Multinomial Mixtures (DMM) and Pachinko Allocation Machine (PAM) models to our dataset. Both the Calinski-Harabasz (CH) index and Silhouette coefficient validated that cluster into two groups were optimal (Figure 4**C**, Fig. S4A, S4B). PAM clusters exhibited different taxonomic drivers: Cluster 1 was characterized by species of *Neisseria* and *Haemophilus*, while Cluster 2 was dominated by species of *Prevotella* and *Veillonella* (Supplemental Table 5), consistent with previously described two predominant oral microbiome stomatotypes 16. Moreover, chi-squared tests confirmed significant OT and stomatotypes associations (P = 0.019), with OT-I samples enriched in Cluster 2 and OT-II in Cluster 1 (Figure 4**C**, Supplemental Table 6). These findings collectively suggest that occlusal type may drive stomatotypes formation through selective enrichment of signature microbiota.

### Distinct interspecies interactions network existed in different OT groups

Uncovering the intricate interspecies interactions in different OT groups will contribute to understand the oral microbiome. Spearman correlation-based co-occurrence networks (| ρ |> 0.8, FDR-adjusted *P* < 0.05) revealed distinct interaction patterns between OT groups (Figure 4**D**).

In OT-I networks, *Prevotella* species (*e.g.*, *P. pallens*) and *Veillonella* species (*e.g.*, *V. atypica*) formed a tightly interconnected cluster (Figure 4**D**). The broad positive correlations among *Prevotella* and *Veillonella* species suggested a potential mutualistic relationship between them.

In contrast, the OT-II group presented two major clusters, which were dominated by *Neisseria* (*e.g.*, *N. flavescens*) and *Haemophilus* species (*e.g.*, *H. parainfluenzae*), respectively (Figure 4**D**). Positive correlations within these clusters and significant negative correlations between *Veillonella montpellierensis* and *Neisseria* species were presented (Figure 4**D**). These results indicated potential support within the same cluster and the competitive dynamics between clusters enriched in different stomatotypes.

Comparative analysis of SparCC networks revealed distinct network topological properties in the OT-I and OT-II networks (Figure 4**E**). The OT-I network presented higher modularity, betweenness centralization, graph diameters and average path lengths (Fig. S5), indicating a more compartmentalized network. In contrast, the OT-II network showed greater complexity and a higher number of interactions, with more edges, greater graph density, average degrees, clustering coefficient and degree centralization. suggesting adaptive adjustment to environmental pressures.

Together, these findings may reveal the potential correlation among the microbial communities and deepen our understanding of the formation and stabilization of different oral microbiome stomatotypes.

### The oral microbiomes of OT-Is and OT-IIs exhibit distinct functional characteristics

**Figure 5:**
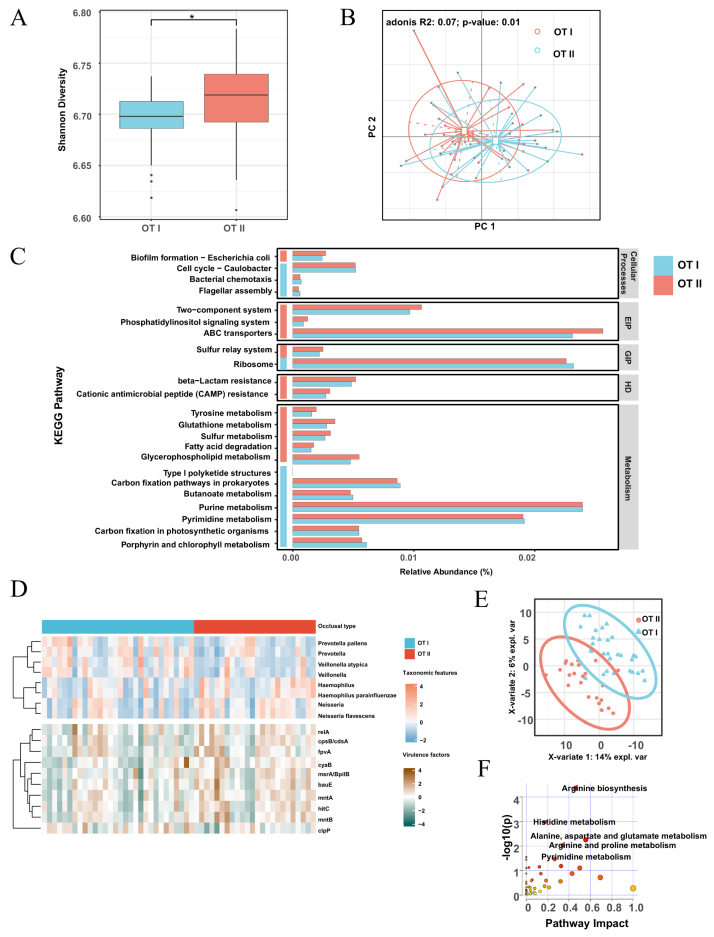
Functional differences in the oral microbiome between OT-I and OT-II groups. **(A)** Comparison of Shannon index revealed functional differences in the oral microbiome between OT-I and OT-II groups. **(B)** PCA analysis presented the distinct difference in gene function between the two groups. **(C)** LEfSe identified differential KEGG pathways between OT-I and OT-II, with an LDA score threshold of 2. **(D)** Heatmap presented representative genera, core species, and the top 10 virulence factor genes from top to bottom. **(E)** The metabolomics were different between OT-I and OT-II groups. **(F)** The enrichment of 5 pathways was revealed by Metabolic pathway analysis (MetPA, P < 0.05).

Functional profiling via the Kyoto Encyclopedia of Genes and Genomes (KEGG) revealed 263 third-level and 42 second-level categories (Supplemental Table 7). OT-II group exhibited a higher Shannon index for gene function than OT-I group, suggesting greater functional diversity (Figure 5**A**). PCA further revealed significant differences between the groups (R ${}^{2}$ : 0.07; P = 0.01) (Figure 5**B**). LEfSe analysis identified 23 OT-associated KEGG pathways (LDA > 2, P < 0.05), with 11 OT-I enriched and 12 OT-II enriched pathways (Figure 5**C**, Fig. S6).

OT-I group enriched pathways major belong to metabolism and cellular processes, such as carbon fixation-related pathways and flagellar assembly pathway. The former likely reflects the predominance of anaerobic bacteria, such as *Prevotella* and *Veillonella*. While the latter may account for the abundance of the *Prevotella*, which is the only one equipped with a flagellar system among the top five genera (Figure 5**C**, Figure 2**B**). Additionally, pathways related to butyrate metabolism, which are beneficial for energy and fat metabolism and provide protection against atherosclerosis 17, 18, also enriched in OT-I groups.

In group OT-II, we observed an enrichment of “Environmental information processing” (EIP) pathways, including the two-component system, phosphatidylinositol signaling system, and ABC transporters; as well as “Human Diseases” (HD) pathways, including beta-Lactam resistance and cationic antimicrobial peptide (CAMP) resistance (Figure 5**C**). These pathways collectively enhance biofilm formation, and drug and peptide resistance 19, 20. Furthermore, OT-II exhibited virulence factor enrichment, such as relA, fpvA, hitc, which showed negative correlations with *Prevotella* and *Veillonella* but positive associations with *Neisseria* and *Haemophilus* (Figure 5**D**). RelA plays an important role in bacterial survival and virulence 21. Similarly, fpvA is pivotal for bacterial growth and proliferation 22. Collectively, these results suggested a higher abundance of potential adverse gene functions in the OT-II group.

### Microbial associated metabolites can be influenced by occlusal type

Microbial metabolites serve as critical mediators of host-microbe crosstalk 23. Given the taxonomic and functional divergence between OT-I and OT-II microbiomes, we hypothesized potential metabolites differences. OPLS-DA analysis confirmed significant metabolomic divergence (Figure 5**E**), with 44 important metabolites identified (P < 0.05, VIP > 1, Supplemental Table 8).

The metabolomic analysis revealed a significant enrichment of metabolites in the OT-II groups, which were predominantly categorized as amino acids (*e.g.*, 4-imidazoleacetate), lipids (*e.g.*, mevalonolactone, 3-hydroxydecanoate), and nucleotides (*e.g.*, N1-methyladenosine, urate,). In contrast, OT-I showed selective enrichment of six metabolites: primarily dipeptides (*e.g.*, phenylalanylglycine, tyrosylglycine) and nucleotides (*e.g.*, uracil, adenosine).

Metabolic pathway analysis (MetPA) revealed the enrichment of five pathways in the two groups (Figure 5**F**), including “histidine metabolism”, “alanine, aspartate and glutamate metabolism”, “pyrimidine metabolism”, “arginine biosynthesis” and “arginine and proline metabolism” (p < 0.05, pathway impact > 0.17). These alterations suggest occlusal type influences microbial metabolism, potentially impacting oral and systemic health.

### Integrated multi-omics analysis uncovered the microbiome-functional pathways-metabolites association

**Figure 6:**
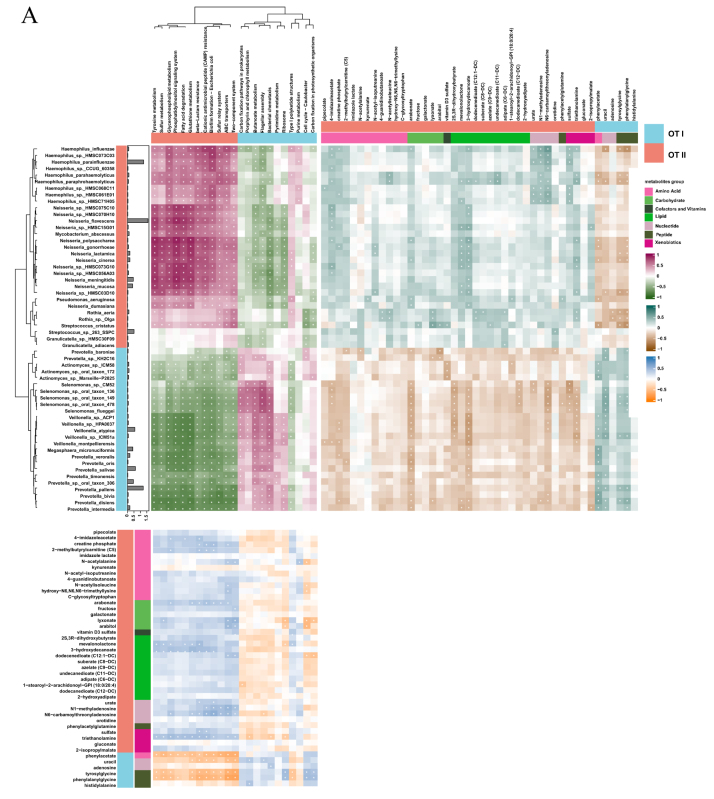
Correlations among differential oral microbiotas, functional pathways, and metabolites. Top left: Heatmap of the Spearman’s rank correlation coefficient between the biomarker species and associated functional pathways. The histogram presented the relative abundance of these differential species. Top right: Heatmap of the Spearman’s rank correlation coefficient between biomarker species and metabolites. Lower left: Heatmap of the Spearman’s rank correlation coefficient between the functional pathways and metabolites. (*P < 0.05).

To elucidate the interconnections among oral microbes, functional pathways, and metabolites, we conducted an integrated analysis. Cross-correlation matrices demonstrated strong intra-group synergy and inter-group antagonism. For example, OT-II enriched taxa such as *H. influenzae*, and *N. flavescens* both positively correlated with ABC transporters but negatively associated with butanoate metabolism, while OT-I signature species such as *P. pallens*, and *V. atypica* exhibited inverse relationships (Figure 6**A**, top left). Correspondingly, this pattern extended to metabolites and differential oral microbes (Figure 6**A**, top right), as well as metabolites and functional pathways (Figure 6**A**, lower left).

**Figure 7:**
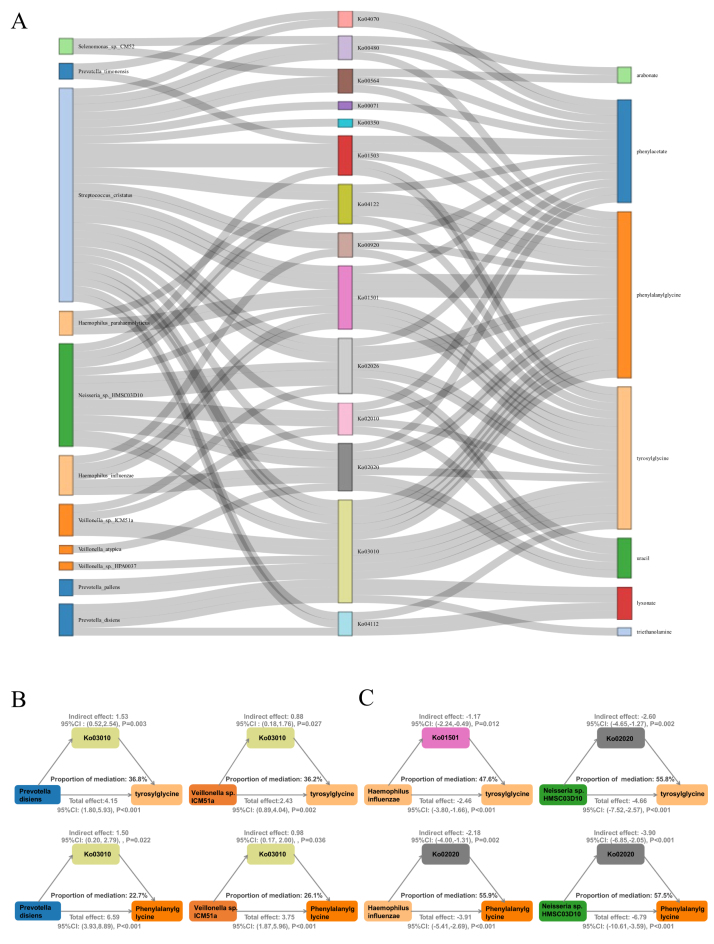
Dipeptides are important metabolites regulated by the oral microbes through differential functional pathways. **(A)** Sankey plot presents the connections between differential oral microbiota, functional pathways and metabolites as identified by mediation analysis (with P value for indirect, direct and total mediation analysis all < 0.05). **(B)** Analysis showing the positive impact of *Prevotella* and *Veillonella* species on the abundance of tyrosylglycine and phenylalanylglycine, mediated by Ko03010. **(C)** Analysis showing the negative impact of *Haemophilus* and *Neisseria* species on the abundance of tyrosylglycine and phenylalanylglycine, mediated by pathway Ko01501 or Ko02020.

Mediation analysis identified 64 significant microbial-metabolite linkages mediated by functional pathways (P values of indirect effect, direct effect and total effect < 0.05) (Figure 7**A**, Supplemental Table 9). Notably, the dipeptides phenylalanylglycine and tyrosylglycine are the most connected metabolites (39/64). Moreover, phenylalanylglycine and tyrosylglycine showed positive associations with *Prevotella* and *Veillonella* species (Figure 7**B**), and negative associations with OT-II-enriched microbes (Figure 7**C**) through different pathways.

Overall, these results suggested a complex interplay with positive correlations within group components and inverse patterns across different groups. Additionally, dipeptides may be an important metabolite in the oral microbiome.

## DISCUSSION

Emerging evidence suggests malocclusion may disrupt oral microbiome homeostasis, while how malocclusion affects the oral microbiome remain unclear. Our study uncovered that occlusion type significantly influences the composition and function of the oral microbiome. Specifically, OT emerged as a driver for the establishment of the “*Prevotella-Veillonella*” and “*Neisseria-Haemophilus*” stomatotypes. The unique microbial interactions network, topological structure, gene functions and multi-omics analysis suggest that different OT associated oral microbiome are not only microbial aggregates, but also functional units with group specific intricate interactions among oral microbes, functional pathways and metabolites.

The rapid development of metagenomics has unveiled the complexity of oral microbiome. According to the Human Microbiome Project (HMP), the oral cavity harbors at least nine distinct microbial habitats, including supragingival plaque, subgingival plaque, saliva et al. 24. Although most of the major oral microbes can be detected in different habitats. However, some oral microbes are specialized for individual niches 24. In this context, saliva emerges as a pragmatic sampling choice for population-level studies due to its non-invasive collection protocol, compatibility with self-sampling, and capacity to integrate microbial signals from multiple oral niches through constant fluid exchange. In this study, we standardized sampling using unstimulated saliva. Unlike stimulated saliva (which alters pH and flow rate), unstimulated sampling not only minimizes exogenous confounders and preserves native saliva sample, but also provides a relatively systemic perspective of oral microbial communities. Further targeted studies of localized pathologies (*e.g.*, periodontitis or dental caries) may require complementary analysis of habitat-specific samples such as subgingival or supragingival plaque. This strategy would enable precise dissection of microbiome-disease relationships within their local microenvironments.

Oral microbial communities influenced by microenvironment, include the surface characteristics of the substrate, gradients of oxygen, and nutrients and proximity to salivary glands 24, 25. In our study, *Prevotella* and *Veillonella*, prevalent in OT-I group, are anaerobes, whereas *Neisseria* and *Haemophilus*, enriched in OT-II group, are aerobes or facultative anaerobes. OT-II-associated traits like increased overjet (5.15 ± 2.70 mm vs. 3.50 ± 1.13 mm in OT-I) and lip incompetence (Figure 1) 26, 27 may elevate air influx, and create a more oxygenated oral environment, which may convert oral microbial communities by rebalancing the ratio of anaerobic and aerobes.

Previous studies have linked drinking water composition to stomatotypes variation, while the specific water components affecting core bacteria such as *Prevotella* and *Neisseria* are remained unclear 16. Interestingly, dipeptides commonly found in surface water 28, and some dipeptides promote the growth of bacteria like *Prevotella*^29^. Our research uncovered that *Prevotella* or *Veillonella* species showed positive correlations with dipeptides, whereas *Neisseria* and *Haemophilus* species exhibited inverse associations. We hypothesize that dipeptides may function as a “bridge” between drinking water and oral microbiome composition.

Oral microecology can also be influence by the relatively long introduction of orthodontic appliances. Recent reviews presented that fixed appliance, significantly impact the oral microenvironment by increasing dental plaque retention sites and hindering the natural self-cleaning and flushing effects of saliva. These changes often promote an increase of gingivitis and caries, especially in the early stage. Removable clear aligners typically exert a milder disturbance due to better accessibility for oral hygiene 30. Our study presented that significant microbial and metabolic differences already exist between different OTs even before the treatment. These baseline microbial signatures may attribute to more personalized preventive interventions. However, further research on how different “stomatotypes” response to orthodontic therapy and how to better support the restoration of microbial balance post-treatment is still required.

There are some limitations in our research. Even though the rarefaction curves confirmed adequate sampling depth for capturing dominant taxa (Fig. S2B, S2C), the cohort size (n=54), while statistically powered based on prior oral-microbiome studies 31, 32, may insufficiently represent rare microbial species or subtle ecological shifts. Furthermore, the restriction to the OT-I/II limits generalizability to broader populations and more complex malocclusions (*e.g.*, OT-III). Second, the function of the dipeptide still needs confirmation with more experiment. To address these gaps, we will establish larger cohorts sample sizes with comprehensive baseline information and oral condition records to further validate and expand these findings. And we will further apply the in vitro and in vivo models testing whether dipeptide supplementation drives oral stomatotypes divergence. Thirdly, for the sampling procedure, 1-hour fasting windows are feasible and provide high-quality samples, overnight fasting might further optimize the concentration of analytes 16, 33. We required a 2-hour fasting and oral hygiene restriction to balance the minimize exogenous interference and goof participant compliance for the adolescent. We cannot entirely exclude the influence of long-term dietary or hygiene habits. Future studies with even more fasting or tooth brushing durations could provide deeper insights into optimizing the sampling procedure.

## MATERIALS AND METHODS

### Study design and participants enrollment

This research was approved by the Institutional Ethics Committee of Shanghai Ninth People’s Hospital, Shanghai Jiao Tong University, School of Medicine (SH9H-2019-T191-6). Fifty-four participants were recruited in this study. Inclusion criteria: aged between 11 and 13 with permanent dentition, occlusal type I or II, without orthodontic treatment. Exclusion criteria: infectious or systemic diseases, history of smoking, alcohol consumption, or antibiotic use within 3 months, visible dental calculus and bleeding on probing gingiva, inability to cooperate with examination and sample collection.

Participants and their parents were well informed about the experimental procedures, potential risks, and benefits, and provided written informed consent before sample collection. Demographic and oral structural assessments were conducted by senior orthodontists at Shanghai Ninth People’s Hospital and verified by another orthodontist before recording. Participants refrained from drinking, eating, or brushing teeth for 2 hours before sample collection. About 2ml saliva was collected using the unstimulated drooling method. Then, saliva stored in an icebox and transferred to the liquid nitrogen tank within 20 min after repackaging and labelling.

### The information of participants

According to previous reports 9–13, The buccal groove of the mandibular first molar occluded by the mesial buccal cusp of the maxillary first molar was defined as OT-I, while the buccal groove positioned distal to the buccal cusp was defined as OT-II. OT-II always company with relative deep overbite and deep overjet.

Basic information, including age, sex, height, weight, body mass index (BMI) and decayed, missing, and filled teeth (DMFT), were recorded. Comprehensive details are provided in Supplemental Table 1–3

### Shot-gun metagenomic sequencing and sequence data processing

Genomic DNA was isolated by Novogene Bioinformatics Technology Co., Ltd. according to the standard procedure. The DNA degradation was assessed in agarose gels. DNA concentration was quantified by the Qubit 2.0 Flurometer (Life Technologies), the OD value is between 1.8 ∼ 2.0, and DNA content over 1 μ g was used for library construction. Next, each qualified DNA sample was fragmented into about 350 bp fragments by ultrasonic crusher. Then, End-polished, A-tailed, and full-length adaptors were then added for further sequencing and PCR amplification. Further, the libraries were purified (AMPure) and the insert size was checked with an Agilent 2100 Bioanalyzer. The concentration of the libraries was then quantified by qPCR. Finally, after clustering, the library preparations were sequenced on the Illumina HiSeq platform and paired-end reads were generated. Closed-source codes were used to cut adapters. The low-quality sequences were removed from the raw data using Readfq (V8, https://github.com/cjfields/readfq). The data was blasted to the host database using Bowtie2. SOAP denovo and MEGAHIT were used for mixed assembly and the Scaftigs ( ≥ 500 bp) were assembled from both single and mixed samples. The contigs predicted the ORF by MetaGeneMark software. Gene function was annotated using mmseqs2 34 based on the KEGG databases.

### Untargeted metabolomics analysis

All the saliva samples were analyzed by the ultra-high performance liquid chromatography-tandem mass spectrometry (UPLC-MS/MS) (Calibra (DIAN) Laboratory) at Calibra Diagnostics in Hangzhou, China, by the Discovery HD4™ Metabolomics Platform. Samples were prepared using an automated MicroLab STAR® system (Hamilton). Proteins were precipitated with methanol under vigorous shaking for 2 minutes (Glen Mills GenoGrinder 2000), followed by centrifugation. Samples were briefly placed on a TurboVap® (Zymark) to remove the organic solvent. Finally, the samples were used for untargeted metabolomics analysis and several types of controls were analyzed in concert with the experimental samples.

### Bioinformatics analysis

The permutation multivariate analysis of variance (PERMANOVA) was conducted based on Bray-Curtis distance in the “vegan” package (version 2.6-4). PCoA based on Bray-Curtis dissimilarity matrices at the genus level were then performed for visualization. The oral microbial species were compared using linear discriminant analysis effect size (LEfSe) 35. The abundances of microbial functional pathways and KEGG pathways were compared using LEfSe. Jensen-Shannon Divergence (JSD) was used to produce distance matrices for samples, and then Partitioning Around Medoids (PAM) clustering was used to group samples with similar overall saliva microbiomes. The Calinski-Harabasz (CH) index and Silhouette index were used to determine the optimal number of clusters. Between-class analysis (BCA) was performed to support the clustering and identify the drivers of the stomatotypes. Analysis and visualization were performed using R with the packages “ade4” (version 1.7-22), “cluster” (version 2.1.4), “clusterSim” (version 0.50-1) and “fpc” (version 2.2-9).

The orthogonal partial least squares discriminant analysis (PLS-DA) was performed using the “ropls” (version 1.26.2) package in R. The online platform, MetaboAnalyst 5.0 (https://www.metaboanalyst.ca/) 36, was used for the MetPA based on untargeted metabolites. Metabolite set enrichment analysis (MSA) was performed using MetaboAnalyst 5.0, based on the pathway-associated metabolite set library 37.

The virulence factors were identified based on the Virulence Factors of Pathogenic Bacteria Database using the Diamond software. Amino acid sequences were aligned against the databases using BLASTP and assigned to genes by the highest-scoring annotated hit of the query protein. The correlation heatmap was generated using the “ComplexHeatmap” package (version 2.14.0) in R. Mediation analyses were performed to evaluate the effects of the KEGG pathways on the associations between the oral microbiome and metabolism. Mediation analyses partitioned the total effect of the oral microbiome on the metabolites into a direct effect of exposure and a mediation effect that accounted for mediators. Two linear models were fitted, one modeling the exposure–mediator association and the other modeling the mediator–outcome association. The mediation effects and the proportion mediated were quantified. The result of the mediation analyses met the following requirements: a) the total effect must be significant; b) the mediation effect must be significant and was visualized with a Sankey plot. This analysis was performed using the packages “mediation” (version 4.5.0) and “Network3D” (version 0.4).

### Statistical analyses

Statistical analyses were performed with R (https://www.r-project.org) and SPSS (version 22.0). Student’s t-test (two-tailed) or Wilcox test and chi-squared test were used to compare the continuous and binary variables between OT-I and OT-II groups, respectively. Alpha diversity indexes were analyzed by Wilcoxon rank-sum test. The difference of representative genera, core species, and the top 10 virulence factor genes between two groups were compared with Wilcox test, and the result of the comparison was presented by z-scores. * Represent p < 0.05; ** represent p < 0.01. As footnotes of tables that show data as mean ± SD.

## AUTHORS CONTRIBUTIONS

**Yufeng Duan:** Contributed to conception and design, data acquisition, analysis and interpretation, drafted and critically revised the manuscript. **Zheng Liu:** Contributed to conception, data acquisition, analysis and interpretation, drafted and critically revised the manuscript. **Wenting Lu:** Contributed to conception, data acquisition, analysis and interpretation, critically revised the manuscript. **Ning Zhao:** Contributed to design, data acquisition, and critically revised the manuscript. **Lingjun Yuan:** Contributed to conception, data acquisition, and critically revised the manuscript. **Zhenxia Li:** Contributed to conception, data acquisition, and critically revised the manuscript. **Ting Zhou:** Contributed to conception, data acquisition, and critically revised the manuscript. **Xiaowen Zheng:** Contributed to conception, data acquisition, and critically revised the manuscript. **Shengzhao Xiao:** Contributed to conception, data acquisition, and critically revised the manuscript. **Dian Jing:** Contributed to conception, data acquisition, and critically revised the manuscript. **Wentao Shi:** Contributed to design, data analysis, and critically revised the manuscript. **Chao Liu:** Contributed to conception and design, data acquisition and analysis, drafting and critically revising the manuscript. **Haixia Lu:** Contributed to conception and design, data acquisition, analysis and interpretation, drafting and critically revising the manuscript. **Qiang Feng:** Contributed to conception and design, data acquisition, analysis and interpretation, drafting and critically revising the manuscript. **Bing Fang:** Contributed to conception and design, data acquisition, analysis and interpretation, drafting and critically revising the manuscript. All authors gave their final approval and agree to be accountable for all aspects of the work.

## SUPPLEMENTAL MATERIAL

All supplemental data for this article are available online at http://microbialcell.com/researcharticles/2026a-duan-microbial-cell/.

.

## CONFLICT OF INTEREST

The authors declare no conflicts of interest.

## ABBREVIATIONS

ABC – ATP-binding cassette

BCA – between-class analysis

BMI – body mass index

CAMP – cationic antimicrobial petide

CH – Calinski-Harabasz

dbRDA – distance-based redundancy analysis

DMFT – decayed, missing, and filled teeth

DMM – Dirichlet Multinomial Mixtures

EIP – environmental information processing

FDR – false discovery rate

HD – human diseases

HMP – human microbiome project

JSD – Jensen-Shannon divergence

KEGG – Kyoto Encyclopedia of Genes and Genomes

LDA – linear discriminant analysis effect size

MESA – metabolite set enrichment analysis

MetPA – metabolic pathway

MRPP – multi-response permutation procedure

OPLS-DA – orthogonal partial least squares discriminant analysis

OT – occlusal type

PAM – partitioning around medoids

PCA – principal component analysis

PCoA – principal component analysis

PCR – polymerase chain reaction

PERMANOVA – permutational multivariate analysis of variance

PLS-DA – partial least squares discriminant analysis

qPCR – quantitative PCR

SD – standard deviation

UPLC-MS/MS – ultra-high performance liquid chromatography-tandem mass spectrometry

VIP – variable importance in projection
