# Supplementary material for: Occlusal types shape oral microbiome stomatotypes and metabolic landscapes: A multi-omics perspective on host-microbe interaction
Source: Microb Cell. 2026 Jun 15;13:237–49. doi: 10.15698/mic2026.06.879 (PMC13320636; doi:10.15698/mic2026.06.879)
Supplement: Supplementary file 1 — . [file mic-13-237-s01.pdf]

Supplemental Figures

Figure S1

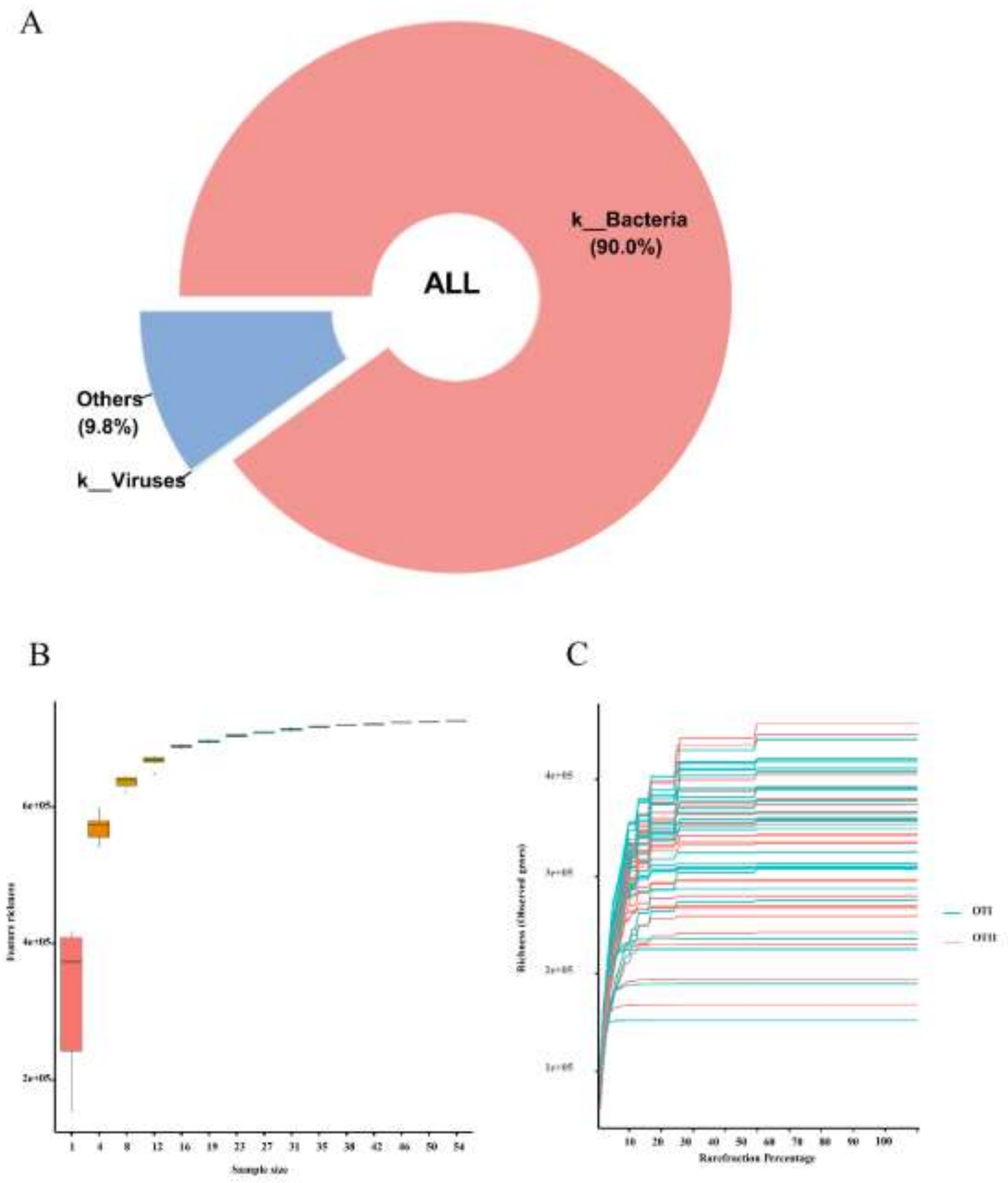

Figure S1. Basic characteristics of metagenomic dates. (A) About 90% of sequences were annotated as bacteria. The feature richness **(B)** and rarefaction curves **(C)** both reached a platform.

Figure S2

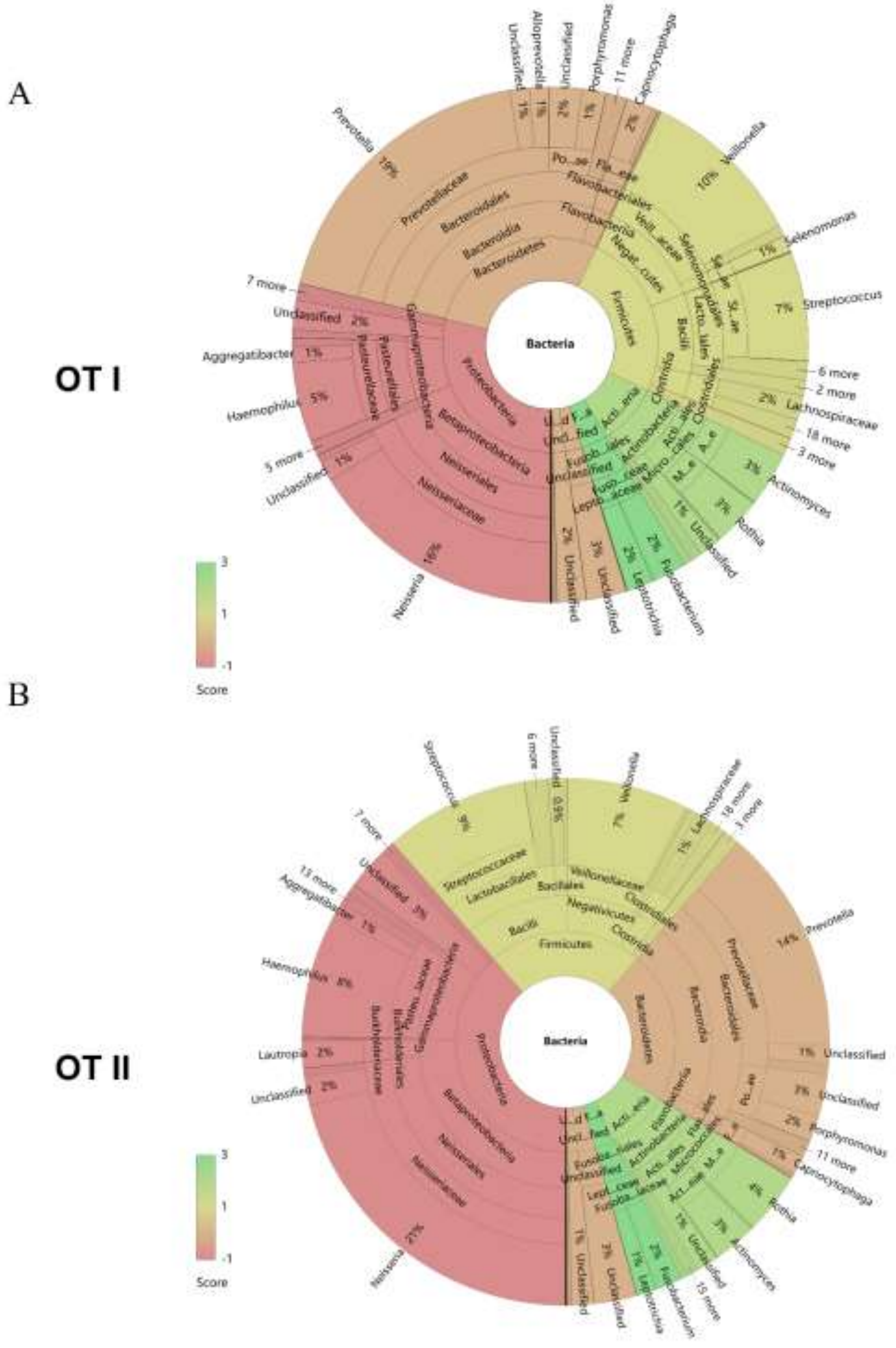

Figure S2. Taxonomic compositions of each group. The major taxonomic compositions of group OT-I **(A)** and OT-II **(B)** were presented respectively.

Figure S3

A

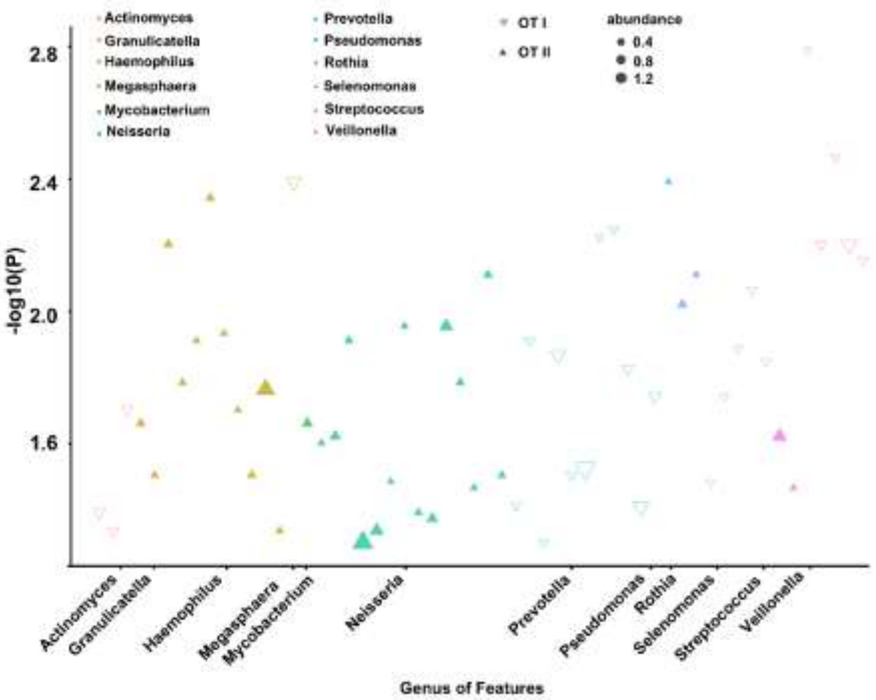

Figure S3 differential species of OT-I and OT-II. (A) Manhattan plot showed differential species distribution to Prevotella, Veillonella genera in the OT-I group and Neisseria, Haemophilus genera in the OT-II group.

Figure S4

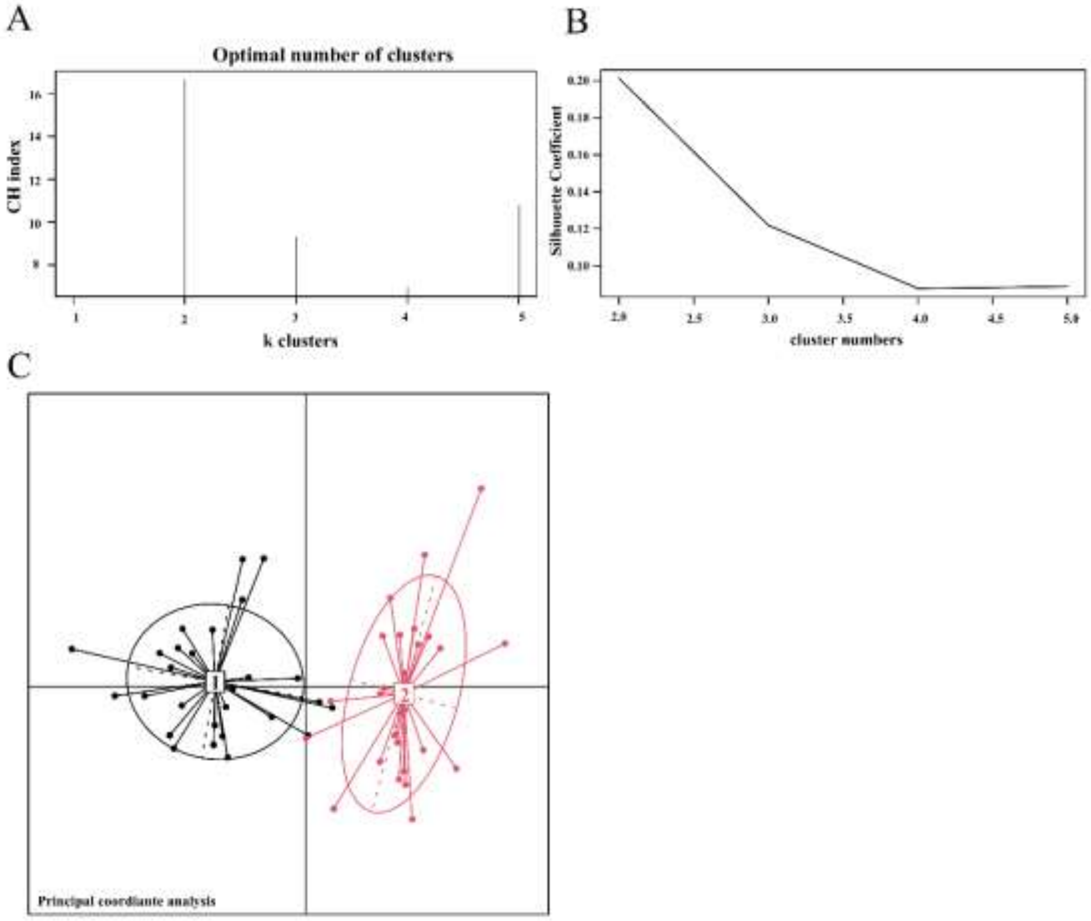

Figure S4 Two distinct oral microbiome stomatotypes can be distinguished in our samples. Both the Calinski-Harabasz (CH) index **(A)** and Silhouette coefficient **(B)** showed that two clusters were the most optimal number of clusters. **(C)** PCA analysis presented an obviously separation exists between these two clusters.

Figure S5

A

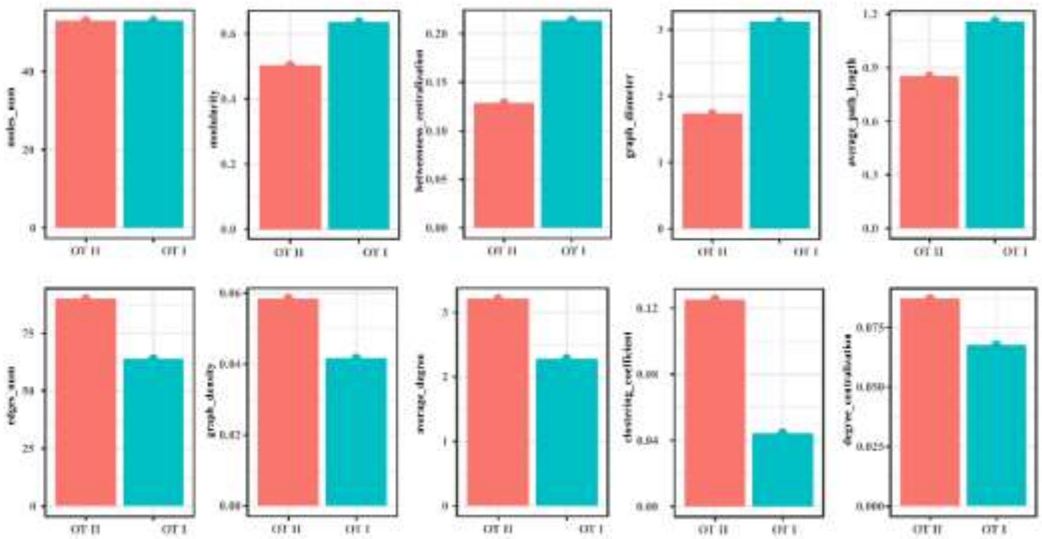

Figure S5 Topological divergence between SparCC networks in OT-I and OT-II microbiota. (A) Presentative topological properties of the SparCC network in OT-I and OT-II groups were presented.

Figure S6

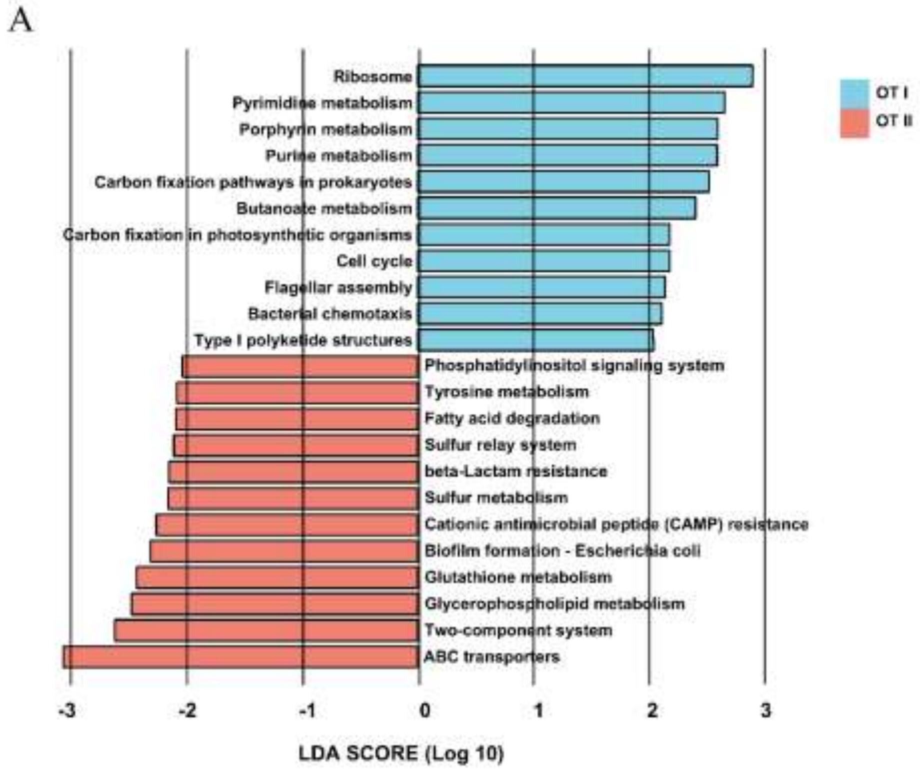

Figure S6 Differential functional pathways were revealed by LEfSe. (A) Twenty-three significantly differential pathways were identified by LEfSe between OT-I and OT-II groups.

Supplemental Tables

Supplemental table 1. The basic information and occlusal parameters of the enrolled participants

	**Participants (N=54)**
Age (Y)	11.61±0.29
Gender	Female: Male=33:21
Height (cm)	155.27±6.99
Weight (kg)	46.24±10.54
BMI (kg/m^2)	19.09±3.97
DMFT	0.22±0.41
Occlusal type	OTI: II=30:24
Crowding at upper teeth (mm)	2.05±2.03
Crowding at lower teeth (mm)	1.42±1.38
Overjet (mm)	4.54±2.29
Overjet: Deep overjet	19:35
Overbite (mm)	4.11±1.48
Overbite: Deep overbite	20:34

Data were presented with mean ± SD

Supplemental table 2. Basic Characteristics of the cohort in OTI and OTII

Number	Height(cm)	Weight(kg)	BMI	Sex	Age(Y)
1	160	39.5	15.43	Female	11.31
2	143	30	14.67	Male	11.93
3	152	40.5	17.53	Male	11.16
4	153	45	19.22	Female	11.65
5	157	58	23.53	Female	12.04
6	147	24	11.11	Female	12.08
7	145	34	16.17	Male	11.43
8	157	36.5	14.81	Female	11.62
9	150	32	14.22	Male	11.63
10	153	43.3	18.5	Male	11.35
11	170	66	22.84	Female	11.8
12	162	65.8	25.07	Male	11.79
13	150	80	35.56	Female	11.64
14	157	47	19.07	Female	11.64
15	154	44	18.55	Female	11.9
16	158	47	18.83	Female	11.67
17	168	45.5	16.12	Female	11.96
18	153	40.1	17.13	Female	11.42
19	156	47	19.31	Male	12.13
20	156	47	19.31	Female	11.62
21	144	34	16.4	Female	11.15
22	155	35	14.57	Female	11.16
23	165	53	19.47	Male	11.22
24	158	58	23.23	Male	11.21
25	158	48.1	19.27	Female	12.04
26	144	61.1	29.47	Female	12.04
27	143	40	19.56	Male	11.33
28	150	36	16	Male	11.37
29	156	56	23.01	Female	11.45
30	160	50	19.53	Male	11.24
31	153	45	19.22	Female	11.48
32	154	42	17.71	Male	11.68
33	155	40	16.65	Female	11.23
34	164	50.8	18.89	Female	12.01
35	156	49	20.13	Female	11.93
36	144	29	13.99	Female	11.75
37	170	53.5	18.51	Male	12.12
38	149	45	20.27	Female	11.59
39	151	46.5	20.39	Male	11.5
40	153	38	16.23	Female	11.13
41	151	32	14.03	Male	11.93
42	160	45	17.58	Female	11.84
43	163	65	24.46	Male	11.47

Number	Height(cm)	Weight(kg)	BMI	Sex	Age(Y)
44	165	50	18.37	Male	11.32
45	145	40	19.02	Female	11.39
46	154	45	18.97	Female	11.53
47	165	50	18.37	Female	11.85
48	152	49.5	21.42	Male	11.42
49	151	39	17.1	Female	12
50	161	42	16.2	Female	11.62
51	147	46	21.29	Male	11.64
52	166	49	17.78	Female	12.1
53	162	62.6	23.85	Female	11.51
54	160	60	23.44	Male	11.36

Supplemental table 3. Oral Characteristics of the cohort in OTI and OTII

Number	Occlusal type	DM FT	Upper crowding (mm)	Lower crowding (mm)	Overjet(mm)	Overbite (mm)
1	I	0	6	4.5	4	3.5
2	I	1	1.5	1	7	6
3	I	1	0	0	4.5	6
4	I	0	0.5	0.5	4	5
5	I	0	0.5	2	2	3
6	I	1	0	1.5	2	5
7	I	0	5	1	6	4.5
8	I	1	4	2	3	5
9	I	0	4.5	1	7	3
10	I	1	0	2	2.5	3
11	I	0	0	0	3.5	4
12	I	0	1	1	3.5	3.5
13	I	0	0	1.5	4	2
14	I	0	0	1	4.5	2.5
15	I	0	2	1.5	3	3.5
16	I	0	5.5	1.5	4	2
17	I	0	3.5	3	10	3.5
18	I	0	1	1	2	4
19	I	0	5	3.5	4.5	3.5
20	I	0	4	1	5	4
21	I	0	0.5	3	4	2.5
22	I	0	3.5	4.5	7	7
23	I	0	0	0	2.5	3.5
24	I	1	0	0	2	2
25	I	0	2	1.5	5	5
26	I	0	0	1.5	3.5	5.5
27	I	0	4.5	0.5	3	3
28	I	0	0	0	3	4
29	I	0	1	0	1.5	2
30	I	0	1.5	0.5	4.5	5
31	II	0	1	1.5	3	6.5
32	II	0	0	0	4.5	4.5
33	II	0	2	0	3	2
34	II	0	5.5	1	2	3
35	II	0	0	0	4.5	3.5
36	II	1	7	2	11	5
37	II	0	3	0	11	6
38	II	1	3	0.5	3	3
39	II	0	2.5	2	9	4.5
40	II	1	1.5	1	6	5
41	II	1	2	2	6	7
42	II	0	3	3.5	3	2
43	II	0	0	0	9	7
44	II	0	4	3	9	6.5
45	II	0	5	5	5	3

Supplemental table 3–Continued

**Number**	**Occlusal type**	**DMFT**	**Upper crowding (mm)**	**Lower crowding (mm)**	**Overjet(mm)**	**Overbite(mm)**
46	II	1	0	1.5	5.5	4
47	II	0	5	1.5	3	2
48	II	0	1	1	4	7
49	II	0	5	1	4	4.5
50	II	1	0	1	3	3
51	II	0	1	1.5	2.5	3
52	II	0	2.5	6	5	6
53	II	0	0	0	4.5	5
54	II	0	0	0	3	3

Supplemental table 4. Chemicals identified by metabolomics

PARENT_SAMPLE_NAME	CHEM_ID	COMP_ID	SUPER_PATHWAY
mevalonate	30	39583	Lipid
1,3-diaminopropane	48	1654	Amino Acid
putrescine	49	1408	Amino Acid
spermidine	50	485	Amino Acid
1-methyladenine	54	1527	Nucleotide
1-methylnicotinamide	55	27665	Cofactors and Vitamins
12,13-DiHOME	62	38395	Lipid
alpha-ketoglutarate	93	528	Energy
kynurenate	98	1417	Amino Acid
3-hydroxyisobutyrate	111	1549	Amino Acid
3-hydroxy-3-methylglutarate	112	531	Lipid
3-phosphoglycerate	132	1414	Carbohydrate
4-hydroxyphenylacetate	144	541	Amino Acid
5,6-dihydrothymine	158	1418	Nucleotide
hypoxanthine	171	3127	Nucleotide
guanine	172	32352	Nucleotide
9,10-DiHOME	179	38399	Lipid
linoleate (18:2n6)	180	1105	Lipid
laurate (12:0)	181	1645	Lipid
N6,N6,N6-trimethyllysine	189	1498	Amino Acid
N-acetylputrescine	192	37496	Amino Acid
N-formylmethionine	194	2829	Amino Acid
adenosine 5'-monophosphate (AMP)	209	32342	Nucleotide
2'-deoxyadenosine	211	1553	Nucleotide
5-methylthioadenosine (MTA)	212	1419	Amino Acid
arachidonate (20:4n6)	229	1110	Lipid
arginine	231	1638	Amino Acid
argininosuccinate	232	15497	Amino Acid
aspartate	234	443	Amino Acid
3-(4-hydroxyphenyl)lactate	240	32197	Amino Acid
phenylpyruvate	241	566	Amino Acid
beta-alanine	244	55	Nucleotide
carnosine	249	1768	Amino Acid
succinate	252	1437	Energy
3-hydroxybutyrate (BHBA)	254	542	Lipid
cholesterol	266	63	Lipid
choline phosphate	267	34396	Lipid
cortisone	273	1769	Lipid
creatinine	275	513	Amino Acid
cystine	279	56	Amino Acid
glucose 6-phosphate	291	31260	Carbohydrate
sphingosine	297	17747	Lipid
dihydroxyacetone phosphate (DHAP)	309	15522	Carbohydrate

Supplemental table 4–Continued

PARENT_SAMPLE_NAME	CHEM_ID	COMP_ID	SUPER_PATHWAY
sphinganine	313	17769	Lipid
flavin adenine dinucleotide (FAD)	327	2134	Cofactors and Vitamins
fumarate	330	1643	Energy
gluconate	338	587	Xenobiotics
glutarate (C5-DC)	339	396	Lipid
glycine	340	58	Amino Acid
guanidinoacetate	344	43802	Amino Acid
2'-deoxyguanosine	348	1411	Nucleotide
histamine	354	1574	Amino Acid
histidine	355	59	Amino Acid
hypotaurine	358	590	Amino Acid
inosine	361	1123	Nucleotide
myo-inositol	363	1124	Lipid
inositol 1-phosphate (I1P)	370	43849	Lipid
isoleucine	376	1125	Amino Acid
2-aminoadipate	381	6146	Amino Acid
3-sulfo-L-alanine	382	47089	Amino Acid
citrulline	391	2132	Amino Acid
leucine	397	60	Amino Acid
lysine	407	1301	Amino Acid
malate	409	1303	Energy
methionine	415	1302	Amino Acid
methylmalonate (MMA)	418	1496	Lipid
nicotinamide	432	594	Cofactors and Vitamins
stearate (18:0)	439	1358	Lipid
ornithine	444	1493	Amino Acid
orotate	445	1505	Nucleotide
palmitoleate (16:1n7)	452	33447	Lipid
phenylalanine	460	64	Amino Acid
phosphate	461	42109	Energy
phosphoenolpyruvate (PEP)	463	597	Carbohydrate
proline	480	1898	Amino Acid
lactate	482	527	Carbohydrate
riboflavin (Vitamin B2)	500	1827	Cofactors and Vitamins
salicylate	501	1515	Xenobiotics
serine	503	1648	Amino Acid
taurine	512	2125	Amino Acid
myristate (14:0)	519	1365	Lipid
urea	533	1670	Amino Acid
uridine	535	606	Nucleotide
2'-deoxyuridine	536	52602	Nucleotide
trans-urocanate	537	607	Amino Acid
glutamate	561	57	Amino Acid
glutamine	563	53	Amino Acid

Supplemental table 4–Continued

PARENT_SAMPLE_NAME	CHEM_ID	COMP_ID	SUPER_PATHWAY
threonine	564	1284	Amino Acid
tryptophan	565	54	Amino Acid
valine	566	1649	Amino Acid
nicotinate	567	1504	Cofactors and Vitamins
pyridoxamine	568	2150	Cofactors and Vitamins
glucose	572	48152	Carbohydrate
adenosine	798	555	Nucleotide
betaine	799	3141	Amino Acid
cysteine	800	1868	Amino Acid
mannose	803	584	Carbohydrate
dimethylglycine	806	5086	Amino Acid
alanine	811	1126	Amino Acid
tyrosine	815	1299	Amino Acid
pseudouridine	821	33442	Nucleotide
pyruvate	823	48990	Carbohydrate
uracil	825	605	Nucleotide
cytidine	827	514	Nucleotide
thymidine	872	2183	Nucleotide
fructose	878	577	Carbohydrate
adenine	880	554	Nucleotide
cytosine	881	573	Nucleotide
thymine	882	604	Nucleotide
caprate (10:0)	888	1642	Lipid
margarate (17:0)	891	1121	Lipid
nonadecanoate (19:0)	892	1356	Lipid
arachidate (20:0)	893	1118	Lipid
maltose	913	15586	Carbohydrate
ribose	914	1471	Carbohydrate
asparagine	917	512	Amino Acid
fucose	918	15587	Carbohydrate
N-stearoyl-sphinganine (d18:0/18:0)*	922	1759	Lipid
dihydroorotate	923	601	Nucleotide
heptanoate (7:0)	925	1644	Lipid
caproate (6:0)	926	32489	Lipid
caprylate (8:0)	932	32492	Lipid
pentadecanoate (15:0)	980	1361	Lipid
trans-4-hydroxyproline	1001	32306	Amino Acid
allantoin	1002	1107	Nucleotide
xanthine	1004	3147	Nucleotide
5-oxoproline	1021	1494	Amino Acid
picolinate	1022	1512	Amino Acid
sarcosine	1023	1516	Amino Acid
pantothenate	1024	1508	Cofactors and Vitamins
pipecolate	1025	1444	Amino Acid

Supplemental table 4–Continued

PARENT_SAMPLE_NAME	CHEM_ID	COMP_ID	SUPER_PATHWAY
phosphoethanolamine	1026	1600	Lipid
glycerate	1052	1572	Carbohydrate
3-ureidopropionate	1053	3155	Nucleotide
N-acetylleucine	1082	1587	Amino Acid
N-acetylmethionine	1083	1589	Amino Acid
N-acetylvaline	1084	1591	Amino Acid
erucate (22:1n9)	1087	1552	Lipid
guanosine	1099	1573	Nucleotide
N-carbamoylaspartate	1108	1594	Nucleotide
N-acetylalanine	1110	1585	Amino Acid
3-aminoisobutyrate	1114	1566	Nucleotide
citrate	1124	1564	Energy
5,6-dihydrouracil	1125	1559	Nucleotide
urate	1134	1604	Nucleotide
valerate (5:0)	1136	33443	Lipid
oleoyl ethanolamide	1137	38102	Lipid
gamma-glutamylglutamine	1140	2730	Peptide
4-hydroxyphenylpyruvate	1141	1669	Amino Acid
butyrate/isobutyrate (4:0)	1143	40605	Lipid
N-acetylneuraminate	1162	32377	Carbohydrate
isocitrate	1206	12110	Energy
N-acetylglucosamine 6-phosphate	1213	15107	Carbohydrate
N-acetylglucosaminylasparagine	1215	48149	Carbohydrate
creatine	1221	27718	Amino Acid
cys-gly, oxidized	1224	18368	Amino Acid
dihomo-linoleate (20:2n6)	1231	17805	Lipid
gamma-glutamylhistidine	1235	18245	Peptide
2-hydroxystearate	1239	17945	Lipid
N1-methyladenosine	1242	15650	Nucleotide
glycerol	1254	15122	Lipid
choline	1256	15506	Lipid
gamma-glutamylleucine	1268	18369	Peptide
N-acetylsphingosine	1302	17772	Lipid
nicotinamide adenine dinucleotide (NAD+)	1310	5278	Cofactors and Vitamins
beta-hydroxyisovalerate	1442	12129	Amino Acid
palmitoyl ethanolamide	1489	38165	Lipid
N-palmitoyl-sphingosine (d18:1/16:0)	1518	44877	Lipid
1-palmitoyl-2-oleoyl-GPE (16:0/18:1)	1526	19263	Lipid
1-palmitoyl-2-oleoyl-GPS (16:0/18:1)	1531	19261	Lipid
1-palmitoyl-2-linoleoyl-GPC (16:0/18:2)	1537	42446	Lipid
stearoyl sphingomyelin (d18:1/18:0)	1538	19503	Lipid
1-palmitoyl-2-oleoyl-GPC (16:0/18:1)	1539	52461	Lipid
azelate (C9-DC)	2029	18362	Lipid
methylsuccinate	2051	15745	Amino Acid

Supplemental table 4–Continued

PARENT_SAMPLE_NAME	CHEM_ID	COMP_ID	SUPER_PATHWAY
tricarballylate	2053	15729	Energy
ethylmalonate	2054	15765	Amino Acid
carnitine	100000007	15500	Lipid
benzoate	100000008	15778	Xenobiotics
3-phenylpropionate (hydrocinnamate)	100000010	15749	Xenobiotics
phenylacetate	100000011	57745	Amino Acid
hippurate	100000014	15753	Xenobiotics
suberate (C8-DC)	100000016	15730	Lipid
3-methyl-2-oxovalerate	100000036	15676	Amino Acid
methionine sulfoxide	100000039	18374	Amino Acid
3-methylhistidine	100000042	15677	Amino Acid
anserine	100000044	15747	Amino Acid
4-guanidinobutanoate	100000096	15681	Amino Acid
agmatine	100000103	15496	Amino Acid
cadaverine	100000110	15308	Amino Acid
creatine phosphate	100000112	33951	Amino Acid
2'-deoxycytidine	100000125	15949	Nucleotide
2'-deoxyinosine	100000135	15076	Nucleotide
flavin mononucleotide (FMN)	100000251	15797	Cofactors and Vitamins
glucuronate	100000257	15443	Carbohydrate
glycerol 3-phosphate	100000258	43847	Lipid
imidazole lactate	100000263	15716	Amino Acid
glycerophosphorylcholine (GPC)	100000269	15990	Lipid
N-acetylglutamate	100000282	15720	Amino Acid
N-acetylmuramate	100000283	18354	Carbohydrate
N-alpha-acetylornithine	100000285	32984	Amino Acid
xanthosine	100000299	15136	Nucleotide
6-phosphogluconate	100000341	15442	Carbohydrate
ribitol	100000406	15772	Carbohydrate
2-isopropylmalate	100000409	15667	Xenobiotics
theophylline	100000437	18394	Xenobiotics
quinate	100000442	18335	Xenobiotics
theobromine	100000445	18392	Xenobiotics
gentisate	100000447	18280	Amino Acid
paraxanthine	100000453	18254	Xenobiotics
5-aminovalerate	100000454	18319	Amino Acid
indolelactate	100000463	18349	Amino Acid
3-indoxyl sulfate	100000467	27672	Amino Acid
glycylvaline	100000487	18357	Peptide
gamma-glutamylphenylalanine	100000491	33422	Peptide
4-methyl-2-oxopentanoate	100000551	22116	Amino Acid
1,5-anhydroglucitol (1,5-AG)	100000580	20675	Carbohydrate
1-stearoyl-2-arachidonoyl-GPI (18:0/20:4)	100000616	52449	Lipid
1-stearoyl-2-oleoyl-GPS (18:0/18:1)	100000639	48494	Lipid

Supplemental table 4–Continued

PARENT_SAMPLE_NAME	CHEM_ID	COMP_ID	SUPER_PATHWAY
1-palmitoyl-2-oleoyl-GPG (16:0/18:1)	100000641	52448	Lipid
1-stearoyl-GPI (18:0)	100000656	19324	Lipid
1,2-dipalmitoyl-GPC (16:0/16:0)	100000657	19130	Lipid
docosahexaenoate (DHA; 22:6n3)	100000665	44675	Lipid
alpha-hydroxyisocaproate	100000706	22132	Amino Acid
isovalerate (i5:0)	100000708	44656	Amino Acid
mevalonolactone	100000772	48118	Lipid
3-hydroxyoctanoate	100000773	22001	Lipid
phenyllactate (PLA)	100000774	22130	Amino Acid
hexanoylcarnitine (C6)	100000781	32328	Lipid
N-acetylaspartate (NAA)	100000787	22185	Amino Acid
dehydroepiandrosterone sulfate (DHEA-S)	100000792	32425	Lipid
acetylcarnitine (C2)	100000802	32198	Lipid
isocaproate (i6:0)	100000806	22273	Lipid
cysteine s-sulfate	100000808	22176	Amino Acid
adipate (C6-DC)	100000863	21134	Lipid
saccharin	100000870	21151	Xenobiotics
1-oleoylglycerol (18:1)	100000924	21184	Lipid
3-methyl-2-oxobutyrate	100000936	44526	Amino Acid
homoarginine	100000961	22137	Amino Acid
triethanolamine	100000989	22202	Xenobiotics
3-hydroxydecanoate	100000997	22053	Lipid
N-acetylglycine	100001006	27710	Amino Acid
ribonate	100001007	27731	Carbohydrate
threonate	100001022	27738	Cofactors and Vitamins
galactonate	100001026	27719	Carbohydrate
indoleacetate	100001034	27513	Amino Acid
1-linoleoylglycerol (18:2)	100001040	27447	Lipid
1-methylhistidine	100001051	30460	Amino Acid
butyrylcarnitine (C4)	100001054	32412	Lipid
isobutyrylcarnitine (C4)	100001055	33441	Amino Acid
2-pyrrolidinone	100001081	31675	Amino Acid
indolepropionate	100001083	32405	Amino Acid
trigonelline (N'-methylnicotinate)	100001092	32401	Cofactors and Vitamins
dodecanedioate (C12-DC)	100001102	3238 8	Lipid
glutamate, gamma-methyl ester	100001103	33487	Amino Acid
N-acetyltyrosine	100001104	32390	Amino Acid
3-hydroxylaurate	100001112	32457	Lipid
threonylphenylalanine	100001125	31530	Peptide
O-acetylhomoserine	100001129	31539	Amino Acid
2-hydroxyadipate	100001153	31934	Lipid
propionylcarnitine (C3)	100001162	32452	Lipid
3-carboxy-4-methyl-5-propyl-2-furanpropanoate (CMPF)	100001178	31787	Lipid
docosadienoate (22:2n6)	100001182	32415	Lipid

Supplemental table 4–Continued

PARENT_SAMPLE_NAME	CHEM_ID	COMP_ID	SUPER_PATHWAY
myristoleate (14:1n5)	100001198	32418	Lipid
4-imidazoleacetate	100001207	32349	Amino Acid
1-methyl-4-imidazoleacetate	100001208	32350	Amino Acid
sebacate (C10-DC)	100001211	32398	Lipid
daidzein	100001219	32453	Xenobiotics
genistein	100001221	32448	Xenobiotics
N-acetylglutamine	100001253	33943	Amino Acid
N-acetyltryptophan	100001254	33959	Amino Acid
N-acetylphenylalanine	100001256	33950	Amino Acid
N-acetylasparagine	100001257	33942	Amino Acid
glycylleucine	100001258	34398	Peptide
gamma-glutamyl-epsilon-lysine	100001262	33934	Peptide
1-palmitoyl-GPC (16:0)	100001263	33955	Lipid
N-acetylarginine	100001266	33953	Amino Acid
1-stearoyl-GPC (18:0)	100001271	33961	Lipid
1-oleoyl-GPC (18:1)	100001272	48258	Lipid
N-acetylthreonine	100001274	33939	Amino Acid
N-acetylisoleucine	100001276	33967	Amino Acid
10-heptadecenoate (17:1n7)	100001278	33971	Lipid
N-acetylhistidine	100001293	33946	Amino Acid
stachydrine	100001296	34384	Xenobiotics
alpha-hydroxyisovalerate	100001300	46537	Amino Acid
nicotinamide riboside	100001310	33013	Cofactors and Vitamins
gamma-glutamylmethionine	100001313	44872	Peptide
gamma-glutamylthreonine	100001314	33364	Peptide
p-cresol sulfate	100001315	36103	Xenobiotics
nicotinate ribonucleoside	100001316	33471	Cofactors and Vitamins
erythronate*	100001320	42420	Carbohydrate
N-acetylproline	100001334	34387	Amino Acid
eicosenoate (20:1)	100001335	33587	Lipid
linolenate [alpha or gamma; (18:3n3 or 6)]	100001337	34035	Lipid
aconitate [cis or trans]	100001359	46173	Energy
isovalerylcarnitine (C5)	100001393	34407	Amino Acid
1-methylxanthine	100001405	34389	Xenobiotics
beta-guanidinopropanoate	100001411	35101	Xenobiotics
N6-carbamoylthreonyladenosine	100001415	35157	Nucleotide
orotidine	100001416	35172	Nucleotide
phenylacetylglutamine	100001417	35126	Peptide
5,6-dihydrouridine	100001425	61833	Nucleotide
3-(3-amino-3-carboxypropyl)uridine*	100001426	62924	Nucleotide
5-methyluridine (ribothymidine)	100001446	35136	Nucleotide
7-methylguanine	100001456	35114	Nucleotide
1-stearoyl-GPE (18:0)	100001461	42398	Lipid
1-stearoyl-GPG (18:0)	100001462	34437	Lipid

Supplemental table 4–Continued

PARENT_SAMPLE_NAME	CHEM_ID	COMP_ID	SUPER_PATHWAY
N1-Methyl-2-pyridone-5-carboxamide	100001468	40469	Cofactors and Vitamins
N1-Methyl-4-pyridone-3-carboxamide	100001469	57584	Cofactors and Vitamins
mead acid (20:3n9)	100001472	35174	Lipid
galactose 6-phosphate	100001498	57615	Carbohydrate
4-hydroxybutyrate (GHB)	100001507	34585	Lipid
2-methylbutyrylcarnitine (C5)	100001509	45095	Amino Acid
phenol sulfate	100001510	32553	Amino Acid
3-ureidoisobutyrate	100001531	57549	Nucleotide
glutamine_degradant*	100001540	46225	Partially Characterized Molecules
2-hydroxy-3-methylvalerate	100001541	36746	Amino Acid
1-palmitoyl-GPE (16:0)	100001567	35631	Lipid
1-oleoyl-GPE (18:1)	100001569	35628	Lipid
1-linoleoyl-GPE (18:2)*	100001570	32635	Lipid
1-arachidonoyl-GPE (20:4n6)*	100001571	35186	Lipid
N-acetylcitrulline	100001577	48434	Amino Acid
2-hydroxypalmitate	100001579	35675	Lipid
gulonate*	100001586	46957	Cofactors and Vitamins
catechol sulfate	100001605	35320	Xenobiotics
cholesterol sulfate	100001606	35324	Lipid
tetradecanedioate (C14-DC)	100001613	35669	Lipid
undecanedioate (C11-DC)	100001617	42395	Lipid
glycerophosphoglycerol	100001619	48857	Lipid
glycerophosphoethanolamine	100001620	37455	Lipid
glycerophosphoinositol*	100001621	52307	Lipid
sedoheptulose-7-phosphate	100001628	35649	Carbohydrate
cyano-alanine	100001645	35660	Amino Acid
deoxycarnitine	100001662	36747	Lipid
1-ribosyl-imidazoleacetate*	100001668	61868	Amino Acid
N2-acetyllysine	100001721	36751	Amino Acid
3,4-dihydroxybutyrate	100001730	62952	Lipid
N6-acetyllysine	100001734	36752	Amino Acid
dihomo-linolenate (20:3n3 or n6)	100001739	35718	Lipid
mannitol/sorbitol	100001740	46142	Carbohydrate
tryptophan betaine	100001743	37097	Amino Acid
methylphosphate	100001805	37070	Nucleotide
dimethylarginine (SDMA + ADMA)	100001810	36808	Amino Acid
N-acetylserine	100001851	37076	Amino Acid
1-stearoyl-2-oleoyl-GPE (18:0/18:1)	100001856	42448	Lipid
1-palmitoyl-2-linoleoyl-GPE (16:0/18:2)	100001870	42449	Lipid
alanylleucine	100001890	37093	Peptide
N-methylproline	100001956	37431	Amino Acid
2-hydroxyglutarate	100002070	37253	Lipid
N-acetyl-beta-alanine	100002102	37432	Nucleotide
5-methylthioribose**	100002103	62100	Amino Acid

Supplemental table 4–Continued

PARENT_SAMPLE_NAME	CHEM_ID	COMP_ID	SUPER_PATHWAY
palmitoyl sphingomyelin (d18:1/16:0)	100002107	37506	Lipid
cysteine sulfinic acid	100002113	37443	Amino Acid
N1,N12-diacetylspermine	100002132	52987	Amino Acid
12-HETE	100002167	37536	Lipid
fructose 1,6-diphosphate/glucose 1,6-diphosphate/myo-inositol diphosphates	100002180	46896	Carbohydrate
13-HODE + 9-HODE	100002196	37752	Lipid
N-acetyl-cadaverine	100002249	43530	Amino Acid
2S,3R-dihydroxybutyrate	100002284	62950	Lipid
(12 or 13)-methylmyristate (a15:0 or i15:0)	100002344	38293	Lipid
(16 or 17)-methylstearate (a19:0 or i19:0)	100002356	38296	Lipid
2R,3R-dihydroxybutyrate	100002367	63109	Lipid
dexpanthenol	100002376	38314	Xenobiotics
alpha-ketoglutaramate*	100002397	62101	Amino Acid
2,3-dihydroxyisovalerate	100002417	38276	Xenobiotics
cysteinylglycine disulfide*	100002466	62103	Amino Acid
formiminoglutamate	100002500	43493	Amino Acid
sulfate*	100002528	46960	Xenobiotics
4-hydroxyglutamate	100002544	40499	Amino Acid
N-methylhydantoin	100002557	40006	Amino Acid
pantoate	100002613	63061	Cofactors and Vitamins
sedoheptulose	100002619	53237	Carbohydrate
2,3-dihydroxy-3-methylvalerate	100002634	63069	Amino Acid
1-methyl-5-imidazoleacetate	100002910	62946	Amino Acid
3-(4-hydroxyphenyl)propionate	100002914	39587	Xenobiotics
S-methylcysteine sulfoxide	100002927	43378	Amino Acid
(14 or 15)-methylpalmitate (a17:0 or i17:0)	100002945	38768	Lipid
UDP-N-acetylglucosamine/galactosamine	100002957	46148	Carbohydrate
1-(1-enyl-palmitoyl)-GPE (P-16:0)*	100003000	39270	Lipid
1-(1-enyl-stearoyl)-GPE (P-18:0)*	100003001	39271	Lipid
isoleucylglycine	100003169	40008	Peptide
leucylalanine	100003179	40010	Peptide
leucylglutamine*	100003183	48189	Peptide
leucylglycine	100003185	40045	Peptide
lysylleucine	100003196	40020	Peptide
valylleucine	100003210	39994	Peptide
2-O-methylascorbic acid	100003258	63990	Cofactors and Vitamins
beta-citrylglutamate	100003271	54923	Amino Acid
trimethylamine N-oxide	100003397	40406	Lipid
N6-methyllysine	100003415	62860	Amino Acid
cis-urocanate	100003425	40410	Amino Acid
imidazole propionate	100003434	40730	Amino Acid
tyrosol	100003493	41515	Amino Acid
histidylalanine	100003542	42027	Peptide
phenylalanylglycine	100003588	41370	Peptide

Supplemental table 4–Continued

PARENT_SAMPLE_NAME	CHEM_ID	COMP_ID	SUPER_PATHWAY
phenylalanylalanine	100003589	41374	Peptide
tyrosylglycine	100003598	41375	Peptide
tryptophylglycine	100003609	43028	Peptide
valylglutamine	100003640	42079	Peptide
valylglycine	100003641	40475	Peptide
prolylglycine	100003674	40703	Peptide
mannonate*	100003695	62864	Xenobiotics
succinimide	100003696	41888	Xenobiotics
3-hydroxyindolin-2-one	100003823	42561	Xenobiotics
(R)-3-hydroxybutyrylcarnitine	100003926	43264	Lipid
histidine betaine (hercynine)*	100004059	62278	Xenobiotics
4-methylcatechol sulfate	100004111	46146	Xenobiotics
lauryl sulfate	100004287	43446	Xenobiotics
2-piperidinone	100004295	43400	Xenobiotics
1-stearoyl-GPS (18:0)*	100004327	45966	Lipid
N-delta-acetylornithine	100004523	43249	Amino Acid
acisoga	100004541	43258	Amino Acid
myristyl sulfate	100004570	44883	Xenobiotics
1-(1-enyl-oleoyl)-GPE (P-18:1)*	100005372	44621	Lipid
fructosyllysine	100005373	46227	Amino Acid
O-sulfo-L-tyrosine	100005384	45413	Xenobiotics
N-acetylmethionine sulfoxide	100005463	45428	Amino Acid
N-acetyltaurine	100005466	48187	Amino Acid
1-palmitoyl-GPG (16:0)*	100005717	45970	Lipid
acetylagmatine	100005945	62698	Amino Acid
2-keto-3-deoxy-gluconate	100005946	48141	Xenobiotics
sphingomyelin (d18:1/24:1, d18:2/24:0)*	100005986	47153	Lipid
N-formylphenylalanine	100006056	48433	Amino Acid
3-hydroxypyridine sulfate	100006098	48448	Xenobiotics
arabonate/xylonate	100006115	48255	Carbohydrate
ribulose/xylulose	100006122	48340	Carbohydrate
N-acetylhistamine	100006123	48679	Amino Acid
behenoyl sphingomyelin (d18:1/22:0)*	100006294	48492	Lipid
lignoceroyl sphingomyelin (d18:1/24:0)	100006298	57330	Lipid
xanthopterin	100006338	54728	Cofactors and Vitamins
3-hydroxyhexanoate	100006367	53230	Lipid
1,2,3-benzenetriol sulfate (2)	100006374	48762	Xenobiotics
3-methoxycatechol sulfate (1)	100006375	48763	Xenobiotics
C-glycosyltryptophan	100006379	48782	Amino Acid
arabitol/xylitol	100006430	48885	Carbohydrate
N-acetylglucosamine/N-acetylgalactosamine	100006435	46539	Carbohydrate
phenol glucuronide	100006679	52747	Amino Acid
1,2-dilinoleoyl-GPC (18:2/18:2)	100008903	52603	Lipid
1-stearoyl-2-oleoyl-GPC (18:0/18:1)	100008904	52438	Lipid
1,2-dioleoyl-GPC (18:1/18:1)	100008905	52457	Lipid

Supplemental table 4–Continued

PARENT_SAMPLE_NAME	CHEM_ID	COMP_ID	SUPER_PATHWAY
1,2-dioleoyl-GPE (18:1/18:1)	100008906	52609	Lipid
1-(1-enyl-stearoyl)-2-oleoyl-GPE (P-18:0/18:1)	100008919	52614	Lipid
2-hydroxybutyrate/2-hydroxyisobutyrate	100008928	52281	Amino Acid
2-methylcitrate/homocitrate	100008929	52282	Energy
oleate/vaccenate (18:1)	100008930	52285	Lipid
palmitoyl dihydrosphingomyelin (d18:0/16:0)*	100008954	52434	Lipid
1-stearoyl-2-linoleoyl-GPE (18:0/18:2)*	100008976	52446	Lipid
1-stearoyl-2-linoleoyl-GPC (18:0/18:2)*	100008980	52452	Lipid
1-oleoyl-2-linoleoyl-GPC (18:1/18:2)*	100008981	52453	Lipid
1-palmitoyl-2-palmitoleoyl-GPC (16:0/16:1)*	100008984	52470	Lipid
alanyl-glutamyl-meso-diaminopimelate	100008987	53165	Peptide
1-(1-enyl-stearoyl)-2-arachidonoyl-GPE (P-18:0/20:4)*	100008999	52475	Lipid
1-(1-enyl-palmitoyl)-2-arachidonoyl-GPE (P-16:0/20:4)*	100009002	52673	Lipid
1-(1-enyl-palmitoyl)-2-oleoyl-GPE (P-16:0/18:1)*	100009005	52477	Lipid
1-(1-enyl-palmitoyl)-2-oleoyl-GPC (P-16:0/18:1)*	100009007	52478	Lipid
1-(1-enyl-palmitoyl)-2-linoleoyl-GPC (P-16:0/18:2)*	100009009	52682	Lipid
sphingomyelin (d18:0/18:0, d19:0/17:0)*	100009027	57473	Lipid
N-palmitoyl-sphinganine (d18:0/16:0)	100009028	52604	Lipid
lactosyl-N-palmitoyl-sphingosine (d18:1/16:0)	100009030	53010	Lipid
1-palmitoyl-2-oleoyl-GPI (16:0/18:1)*	100009066	52669	Lipid
1-(1-enyl-palmitoyl)-2-linoleoyl-GPE (P-16:0/18:2)*	100009069	52677	Lipid
1-oleoyl-2-linoleoyl-GPE (18:1/18:2)*	100009078	52687	Lipid
1-(1-enyl-stearoyl)-2-linoleoyl-GPE (P-18:0/18:2)*	100009225	52748	Lipid
(S)-3-hydroxybutyrylcarnitine	100009271	52984	Lipid
hexadecadienoate (16:2n6)	100009394	57652	Lipid
succinylglutamine	100010853	55014	Amino Acid
2'-O-methylcytidine	100010895	57554	Nucleotide
2'-O-methyluridine	100010896	57655	Nucleotide
2-hydroxybehenate	100015637	57663	Lipid
2-hydroxynervonate*	100015638	61698	Lipid
N,N,N-trimethyl-5-aminovalerate	100015962	57687	Amino Acid
(N(1) + N(8))-acetylspermidine	100016038	57814	Amino Acid
2-hydroxyarachidate*	100019892	61700	Lipid
lyxonate	100019968	61858	Carbohydrate
dodecenedioate (C12:1-DC)*	100019972	61864	Lipid
N,N,N-trimethyl-alanylproline betaine (TMAP)	100020217	62947	Amino Acid
3-formylindole	100020219	62863	Xenobiotics
diacetylspermidine*	100020242	62105	Amino Acid
tetradecadienoate (14:2)*	100020343	62850	Lipid
3-amino-2-piperidone	100020361	62853	Amino Acid
N-acetyl-isoputreanine	100020487	62309	Amino Acid
3-hydroxyhexanoylcarnitine (1)	100020825	62557	Lipid
2,6-dihydroxybenzoic acid	100020837	63042	Xenobiotics

Supplemental table 4–Continued

PARENT_SAMPLE_NAME	CHEM_ID	COMP_ID	SUPER_PATHWAY
2,3-dihydroxy-5-methylthio-4-pentenoate (DMTPA)*	100020893	62805	Amino Acid
Fibrinopeptide A (2-15)**	100020902	62809	Peptide
vitamin D3 sulfate	100021071	64558	Cofactors and Vitamins
hydroxy-N6,N6,N6-trimethyllysine*	100021123	62959	Amino Acid
2-hydroxy-4-(methylthio)butanoic acid	100021372	63739	Amino Acid
pentose acid*	100021467	63264	Partially Characterized Molecules
N-carbamoylputrescine	100021503	63854	Amino Acid
oxindolylalanine	100022120	64568	Amino Acid

Supplemental table 5. The representative driver species of PAM clusters

Taxa	PAM cluster1	PAM cluster2
Prevotella.disiens	-0.674919266	0.909673794
Veillonella.dispar	-0.643974269	0.867965318
Prevotella.fusca	-0.641548443	0.864695727
Prevotella.sp..F0091	-0.635534961	0.856590599
Prevotella.denticola	-0.634929807	0.855774957
Prevotella.jejuni	-0.632118506	0.851985812
Prevotella.bivia	-0.623134535	0.839876982
Veillonella.atypica	-0.617944735	0.832882035
Prevotella.falsenii	-0.613999012	0.827563886
Prevotella.histicola	-0.611027856	0.823559284
Prevotella.sp..C561	-0.607340022	0.818588725
Prevotella.multiformis	-0.602543351	0.812123647
Veillonella.sp..ICM51a	-0.592954335	0.799199321
Veillonella.sp..HPA0037	-0.592709905	0.798869872
Veillonella.sp..ACP1	-0.589940589	0.795137315
Prevotella.oris	-0.586827088	0.790940857
Prevotella.sp..ICM33	-0.582952084	0.785718026
Prevotella.melaninogenica	-0.581440658	0.783680887
Prevotella.scopos	-0.577604129	0.778509912
Prevotella.saccharolytica	-0.570490389	0.768921829
Prevotella.corporis	-0.569978348	0.768231687
Prevotella.nigrescens	-0.569065511	0.767001341
Prevotella.veroralis	-0.568913969	0.766797088
Prevotella.intermedia	-0.565668442	0.762422683
Megasphaera.micronuciformis	-0.55699459	0.750731839
Prevotella.sp..HJM029	-0.550109216	0.741451552
Veillonella.sp..DORA_A_3_16_22	-0.548019484	0.738634957
Atopobium.sp..ICM42b	-0.546464878	0.736539618
Mogibacterium.pumilum	-0.544568216	0.733983248
Veillonella.sp..AS16	-0.541331466	0.729620672
Prevotella.pallens	-0.537177691	0.724022105
Atopobium.parvulum	-0.535871933	0.722262171
Prevotella.oulorum	-0.525712004	0.708568353
Atopobium.sp..BS2	-0.524437649	0.706850744
Prevotella.buccalis	-0.522759929	0.70458947
Veillonella.montpellierensis	-0.507662925	0.684241334
Prevotella.timonensis	-0.507391184	0.683875074
Prevotella.amnii	-0.493428925	0.665056377
Prevotella.multisaccharivorax	-0.485006369	0.653704237
Bacteroides.fragilis	-0.477258264	0.643261139
X.Eubacterium..sulci	-0.47677432	0.642608866
Prevotella.bryantii	-0.473754354	0.638538477
Oribacterium.sp..oral.taxon.108	-0.471838175	0.635955802
Oribacterium.asaccharolyticum	-0.466322683	0.628521877
Bacteroidales.bacterium.KA00344	-0.455310579	0.613679476
Veillonella.rodentium	-0.44958956	0.605968537
Actinomyces.graevenitzii	-0.441212298	0.594677445

Supplemental table 5–Continued

**Taxa**	**PAM cluster1**	**PAM cluster2**
Prevotella.salivae	-0.43178726	0.581974132
Lachnospiraceae.bacterium.oral.taxon.082	-0.422495708	0.569450737
Prevotella.sp..oral.taxon.306	-0.422170235	0.569012056
Stomatobaculum.longum	-0.417440614	0.56263735
Prevotella.maculosa	-0.396726209	0.534717933
Solobacterium.moorei	-0.396406443	0.534286945
Actinomyces.sp..ICM47	-0.383734881	0.517207883
Candidatus.Saccharibacteria.oral.taxon.TM7x	-0.382452532	0.515479499
X.Eubacterium..infirmum	-0.365285552	0.492341396
Rothia.sp..HMSC065C03	-0.359478717	0.484514793
Veillonella.sp..6_1_27	-0.357101345	0.481310508
Prevotella.sp..HUN102	-0.354692172	0.478063363
Actinomyces.sp..HMSC035G02	-0.354479748	0.477777051
Actinomyces.sp..oral.taxon.181	-0.354005722	0.477138148
Alloprevotella.rava	-0.346771172	0.467387231
Clostridium.sp..CAG.793	-0.345500213	0.465674201
Actinomyces.sp..HPA0247	-0.343873508	0.463481685
Isoptericola.variabilis	-0.340510074	0.45894836
Trueperella.pyogenes	-0.337261273	0.454569542
Actinomyces.sp..ph3	-0.333179678	0.449068261
Actinomyces.sp..S6.Spd3	-0.328548943	0.442826836
Rothia.sp..HMSC061D12	-0.317154214	0.427468723
Veillonella.sp..3_1_44	-0.315635683	0.425422008
Sanguibacter.keddieii	-0.312209381	0.420803949
Actinomyces.sp..oral.taxon.172	-0.311358655	0.419657318
Leptotrichia.sp..oral.taxon.847	-0.306262276	0.412788285
Prevotella.sp..CAG.1092	-0.301592102	0.406493703
Actinomyces.sp..ICM58	-0.299886385	0.404194693
Lachnoanaerobaculum.saburreum	-0.296380268	0.399469057
Candidatus.Saccharibacteria.bacterium.47.87	-0.291627548	0.393063217
Selenomonas.flueggei	-0.288682504	0.389093809
Selenomonas.sp..oral.taxon.478	-0.283553999	0.382181476
Veillonella.sp..DORA_B_18_19_23	-0.282951139	0.381368926
Centipeda.periodontii	-0.274636125	0.370161734
Selenomonas.sp..oral.taxon.136	-0.274307677	0.369719043
Selenomonas.sp..CM52	-0.272653646	0.367489696
Selenomonas.sp..oral.taxon.149	-0.271819112	0.36636489
Oribacterium.parvum	-0.265839751	0.358305751
Campylobacter.concisus	-0.264567946	0.35659158
Leptotrichia.goodfellowii	-0.264361615	0.356313481
Leptotrichia.sp..oral.taxon.498	-0.261844132	0.352920352
Alloprevotella.tannerae	-0.257940957	0.347659551
Streptococcus.agalactiae	-0.253872254	0.342175646
Selenomonas.sputigena	-0.251120632	0.338466939
Candidatus.Saccharibacteria.bacterium.32.50.13	-0.24686945	0.332737085
Actinomyces.odontolyticus	-0.245033857	0.330263024
Campylobacter.sp..10_1_50	-0.24338218	0.328036852
Leptotrichia.trevisanii	-0.233969937	0.315350785

Supplemental table 5–Continued

Taxa	PAM cluster1	PAM cluster2
Veillonella.parvula	-0.233368409	0.31454003
Candidatus.Saccharibacteria.bacterium	-0.230637123	0.310858731
Prevotella.copri	-0.223282215	0.300945594
Prevotella.sp..KH2C16	-0.223271941	0.300931747
Selenomonas.infelix	-0.213057052	0.287163852
Streptococcus.parasanguinis	-0.205894419	0.27750987
Leptotrichia.wadei	-0.204904185	0.276175206
Selenomonas.sp..oral.taxon.126	-0.204807725	0.276045195
Streptococcus.salivarius	-0.199382823	0.26873337
Leptotrichia.sp..oral.taxon.879	-0.192761739	0.259809301
Mycoplasma.sp..CAG.877	-0.192557098	0.259533481
Oribacterium.sinus	-0.191600576	0.258244255
Veillonella.sp..CAG.933	-0.176772215	0.238258202
Candidatus.Saccharibacteria.bacterium.32.49.12	-0.17353978	0.233901443
Streptococcus.sp..HPH0090	-0.166789405	0.224803112
Mycobacterium.tuberculosis	-0.164912447	0.222273298
Xylanimonas.cellulosilytica	-0.159468699	0.214936072
Streptococcus.sp..263_SSPC	-0.152061407	0.204952332
Actinomyces.sp..ICM39	-0.148190555	0.199735096
Actinomyces.sp..ICM54	-0.142426383	0.191965995
Candidatus.Saccharibacteria.bacterium.49.20	-0.139589416	0.188142256
Actinomyces.sp..Marseille.P2825	-0.135319851	0.182387625
Leptotrichia.hofstadii	-0.130537623	0.175942014
Actinomyces.oris	-0.108008667	0.145576899
Rothia.dentocariosa	-0.091308592	0.123068102
Prevotella.sp..oral.taxon.299	-0.088306837	0.119022259
Leptotrichia.shahii	-0.080010162	0.107839784
Peptostreptococcus.stomatis	-0.079938685	0.107743446
Candidatus.Saccharibacteria.bacterium.GW2011_GWC2_48_9	-0.078806709	0.106217738
Tannerella.sp..oral.taxon.HOT.286	-0.07635556	0.102914015
Lachnoanaerobaculum.sp..OBRC5.5	-0.075543505	0.101819506
Prevotella.conceptionensis	-0.075327665	0.101528592
Lachnoanaerobaculum.sp..MSX33	-0.070317386	0.094775608
Streptococcus.infantis	-0.065039767	0.087662294
Granulicatella.adiacens	-0.06459432	0.087061909
Pseudomonas.aeruginosa	-0.064210349	0.086544383
Rothia.mucilaginosa	-0.062261522	0.083917704
Catonella.morbi	-0.061919881	0.083457231
Prevotella.sp..oral.taxon.473	-0.061844441	0.083355551
Gemella.sanguinis	-0.05661127	0.076302147
Lachnoanaerobaculum.sp..ICM7	-0.037646174	0.050740496
Prevotella.nanceiensis	-0.033914205	0.04571045
Porphyromonas.gingivalis	-0.017516623	0.023609362
Candidatus.Saccharimonas.aalborgensis	-0.016531565	0.022281674
Pseudopropionibacterium.propionicum	-0.016400546	0.022105083
Veillonella.sp..oral.taxon.158	-0.00900566	0.012138063
Candidatus.Saccharibacteria.bacterium.32.50.10	-0.002384988	0.00321455

Supplemental table 5–Continued

Taxa	PAM cluster1	PAM cluster2
Granulicatella.sp..HMSC31F03	0.000848774	-0.001144
Veillonella.sp..oral.taxon.780	0.004833622	-0.006514882
Kingella.oralis	0.014905943	-0.020090619
Peptoniphilus.lacrimalis	0.015181123	-0.020461514
Veillonella.sp..DNF00869	0.016544921	-0.022299676
Granulicatella.sp..HMSC30F09	0.017301907	-0.023319961
Prevotella.sp..oral.taxon.317	0.024359952	-0.032832979
Leptotrichia.sp..oral.taxon.215	0.03006775	-0.040526098
Rothia.sp..Olga	0.030993447	-0.041773776
Prevotella.sp..HMSC073D09	0.036272277	-0.048888722
Abiotrophia.sp..HMSC24B09	0.045199748	-0.0609214
Prevotella.aurantiaca	0.053175653	-0.071671532
Bacillus.thuringiensis	0.05952561	-0.08023017
Prevotella.loescheii	0.061776251	-0.083263643
Rothia.aeria	0.063107721	-0.085058233
Porphyromonas.catoniae	0.07724192	-0.104108674
Abiotrophia.defectiva	0.079096799	-0.10660873
Escherichia.coli	0.079817091	-0.107579557
Chlamydia.trachomatis	0.082424248	-0.111093552
uncultured.bacterium	0.090687684	-0.122231226
Veillonella.tobetsuensis	0.092510696	-0.124688329
Fusobacterium.hwasookii	0.097091423	-0.130862352
Leptotrichia.buccalis	0.110893529	-0.149465191
Neisseria.sp..N95_16	0.117219969	-0.157992132
Corynebacterium.matruchotii	0.121192516	-0.163346435
Streptococcus.suis	0.12384737	-0.166924716
Leptotrichia.sp..oral.taxon.212	0.131677688	-0.177478623
Bacteroidetes.oral.taxon.274	0.134919204	-0.181847623
Lautropia.mirabilis	0.138209945	-0.18628297
Streptococcus.peroris	0.140913858	-0.189927374
Capnocytophaga.gingivalis	0.149493614	-0.201491393
Fusobacterium.nucleatum	0.155408451	-0.209463565
Gemella.morbillorum	0.159378798	-0.214814901
Ottowia.sp..oral.taxon.894	0.165035053	-0.22243855
Caballeronia.arationis	0.1669052	-0.224959183
Prevotella.sp..oral.taxon.472	0.168162018	-0.226653154
Neisseria.dumasiana	0.172824573	-0.232937468
Porphyromonas.sp..COT.239.OH1446	0.173487908	-0.233831529
Porphyromonas.endodontalis	0.184345202	-0.248465273
Streptococcus.pseudopneumoniae	0.184424869	-0.248572649
Capnocytophaga.leadbetteri	0.184588231	-0.248792833
Moraxella.oblonga	0.186863786	-0.251859885
Moraxella.sp..VT.16.12	0.188953213	-0.25467607
Neisseria.sp..oral.taxon.014	0.192625512	-0.259625691
Capnocytophaga.sp..oral.taxon.329	0.194511226	-0.262167305
Streptococcus.sanguinis	0.196373258	-0.264677
Fusobacterium.periodonticum	0.197264047	-0.265877629
candidate.division.SR1.bacterium	0.198167504	-0.267095332

Supplemental table 5–Continued

Taxa	PAM cluster1	PAM cluster2
candidate.division.SR1.bacterium.RAAC1_SR1_1	0.199929114	-0.269469676
candidate.division.SR1.bacterium.CG_4_9_14_3_um_filter_40_9	0.200120862	-0.269728118
Moraxella.caviae	0.202031521	-0.272303355
Bergeyella.zoohelcum	0.202603093	-0.273073734
Prevotella.shahii	0.203829276	-0.274726416
uncultured.bacterium..gcode.4.	0.205948347	-0.277582554
Staphylococcus.pasteuri	0.205990133	-0.277638875
candidate.division.SR1.bacterium.Aalborg_AAW.1	0.206456964	-0.278268082
Riemerella.anatipestifer	0.206843042	-0.278788447
Parvimonas.micra	0.21008668	-0.283160307
Prevotella.baroniae	0.210171141	-0.283274146
Haemophilus.sp..CCUG.66565	0.213489633	-0.287746897
Capnocytophaga.sp..oral.taxon.332	0.213766769	-0.288120428
Porphyromonas.sp..oral.taxon.278	0.215110677	-0.289931782
Streptococcus.oralis	0.220249043	-0.296857405
Streptococcus.sp..oral.taxon.058	0.221128395	-0.298042619
Prevotellaceae.bacterium.Marseille.P2826	0.223720256	-0.301535997
Fusobacterium.sp..oral.taxon.370	0.230029491	-0.310039749
Kingella.denitrificans	0.23110897	-0.311494699
Haemophilus.sp..HMSC71H05	0.244945176	-0.330143498
Candidatus.Gracilibacteria.bacterium	0.245777358	-0.331265134
Kingella.kingae	0.249144783	-0.335803838
Streptococcus.pneumoniae	0.252940869	-0.340920301
Treponema.sp.	0.254232351	-0.342660995
Capnocytophaga.sp..oral.taxon.863	0.254969271	-0.343654235
Granulicatella.elegans	0.255443698	-0.34429368
Aggregatibacter.segnis	0.25608835	-0.345162558
Streptococcus.mitis	0.257843443	-0.347528119
Campylobacter.showae	0.261843443	-0.352919423
Streptococcus.cristatus	0.262713192	-0.354091693
Haemophilus.sputorum	0.26309983	-0.354612815
Capnocytophaga.sp..oral.taxon.338	0.263418042	-0.355041709
Haemophilus.haemolyticus	0.26426182	-0.356178975
Gemella.haemolysans	0.272584665	-0.367396722
Neisseria.sp..HMSC070A01	0.280865738	-0.378558168
Haemophilus.sp..HMSC068C11	0.289966342	-0.390824201
Capnocytophaga.sp..oral.taxon.326	0.290155947	-0.391079754
Streptococcus.sp..M334	0.292665603	-0.394462334
Haemophilus.sp..HMSC61B11	0.294586699	-0.397051638
Haemophilus.paraphrohaemolyticus	0.300764167	-0.40537779
Capnocytophaga.granulosa	0.302510115	-0.407731025
Capnocytophaga.sp..CM59	0.303620905	-0.409228176
Cloacibacterium.normanense	0.305432924	-0.411670463
Haemophilus.sp..HMSC061E01	0.310224767	-0.418129034
Haemophilus.parahaemolyticus	0.315414174	-0.425123452
Aggregatibacter.aphrophilus	0.317298113	-0.427662674
Capnocytophaga.sputigena	0.317462665	-0.427884462

Supplemental table 5–Continued

Taxa	PAM cluster1	PAM cluster2
Porphyromonas.somerae	0.319291002	-0.430348742
Porphyromonas.sp..KLE.1280	0.328967423	-0.443390874
Neisseria.bacilliformis	0.333617385	-0.449658214
Porphyromonas.sp..oral.taxon.279	0.340010822	-0.458275456
Neisseria.sp..HMSC065D04	0.343520589	-0.463006011
Aggregatibacter.actinomycetemcomitans	0.346410233	-0.466900749
Capnocytophaga.sp..ChDC.OS43	0.348966809	-0.470346568
Neisseria.sp..HMSC70E02	0.360271316	-0.485583078
Neisseria.sp..HMSC31F04	0.361995327	-0.487906746
Aggregatibacter.sp..oral.taxon.458	0.362600656	-0.488722623
Neisseria.sp..HMSC074B07	0.370097384	-0.498826909
Haemophilus.sp..oral.taxon.851	0.375991364	-0.506770969
Haemophilus.parainfluenzae	0.376958069	-0.50807392
Halobacillus.trueperi	0.385436229	-0.519501004
Neisseria.sp..HMSC065C04	0.395670582	-0.533295132
Haemophilus.pittmaniae	0.407729454	-0.549548395
Eikenella.sp..NML130454	0.410058886	-0.552688064
Cardiobacterium.hominis	0.41019982	-0.552878018
Neisseria.sp..HMSC15C08	0.411489179	-0.554615849
Streptococcus.timonensis	0.419057537	-0.56481668
Neisseria.elongata	0.424248479	-0.571813167
Neisseria.cinerea	0.434833059	-0.586079341
Haemophilus.sp..HMSC073C03	0.446262507	-0.601484249
Haemophilus.influenzae	0.448275751	-0.604197752
Haemophilus.sp..CCUG.60358	0.449583069	-0.605959788
Neisseria.sp..HMSC064E01	0.45970979	-0.619608847
Neisseria.macacae	0.462921905	-0.62393822
Neisseria.sp..GT4A_CT1	0.470870771	-0.634651908
Neisseria.subflava	0.47921145	-0.645893694
Neisseria.sp..HMSC078C12	0.488134451	-0.657920347
Neisseria.sp..HMSC06F02	0.488664197	-0.658634352
Neisseria.sp..HMSC068C04	0.489169499	-0.659315411
Mycobacterium.abscessus	0.490233636	-0.660749684
Neisseria.sp..HMSC061B04	0.495072475	-0.667271596
Neisseria.sp..HMSC077D05	0.503793358	-0.679025831
Neisseria.sp..HMSC072F04	0.509944356	-0.687316306
Neisseria.sp..HMSC066H01	0.510401091	-0.687931905
Neisseria.sp..HMSC073B07	0.514933114	-0.694040285
Neisseria.sp..HMSC055H02	0.519682219	-0.700441251
Neisseria.sicca	0.525891693	-0.708810542
Morococcus.cerebrosus	0.527403329	-0.710847965
Neisseria.sp..HMSC064D07	0.531121303	-0.715859147
Porphyrobacter.donghaensis	0.53299394	-0.718383136
Neisseria.sp..HMSC15G01	0.536004699	-0.722441117
Neisseria.sp..HMSC056A03	0.546925024	-0.737159816
Neisseria.sp..HMSC064F03	0.556892625	-0.750594408
Neisseria.sp..HMSC069H12	0.558760326	-0.753111744
Neisseria.sp..HMSC063B05	0.561387295	-0.756652441

Supplemental table 5–Continued

**Taxa**	**PAM cluster1**	**PAM cluster2**
Neisseria.sp..HMSC03D10	0.589798347	-0.794945599
Eikenella.corrodens	0.594361121	-0.801095424
Neisseria.sp..HMSC070H10	0.598730093	-0.806984038
Neisseria.sp..HMSC073G10	0.608381031	-0.819991824
Neisseria.mucosa	0.617641731	-0.832473638
Neisseria.gonorrhoeae	0.619410067	-0.834857047
Neisseria.sp..HMSC075C10	0.620570709	-0.836421391
Acinetobacter.baumannii	0.622639467	-0.839209716
Neisseria.flavescens	0.622975509	-0.839662642
Neisseria.sp..HMSC072C05	0.642454357	-0.865916742
Neisseria.lactamica	0.654613928	-0.882305729
Neisseria.polysaccharea	0.657904684	-0.886741096
Neisseria.meningitidis	0.671237415	-0.904711299

Supplemental table 6 OTIs and OTIIs were mainly classified into different clusters

Sample	PAM Clusters	Occlusal type
A0119-001460	PAM_2	occlusal type I
A0119-001486	PAM_2	occlusal type I
A0119-001490	PAM_2	occlusal type I
A0119-001492	PAM_2	occlusal type I
A0119-001497	PAM_2	occlusal type I
A0119-001542	PAM_2	occlusal type I
A0119-001595	PAM_2	occlusal type II
A0119-001605	PAM_2	occlusal type I
A0119-001609	PAM_2	occlusal type II
A0119-001671	PAM_2	occlusal type I
A0119-001672	PAM_2	occlusal type II
A0119-001676	PAM_2	occlusal type II
A0119-002211	PAM_2	occlusal type I
A0119-002293	PAM_2	occlusal type II
A0119-002326	PAM_2	occlusal type I
A0119-002427	PAM_2	occlusal type I
A0119-002444	PAM_2	occlusal type I
A0119-002518	PAM_2	occlusal type I
A0119-002521	PAM_2	occlusal type I
A0119-002524	PAM_2	occlusal type II
A0119-002892	PAM_2	occlusal type I
A0119-002896	PAM_2	occlusal type I
A0119-002901	PAM_2	occlusal type I
A0119-001459	PAM_1	occlusal type II
A0119-001488	PAM_1	occlusal type I
A0119-001489	PAM_1	occlusal type II
A0119-001499	PAM_1	occlusal type II
A0119-001501	PAM_1	occlusal type I
A0119-001514	PAM_1	occlusal type I
A0119-001525	PAM_1	occlusal type II
A0119-001546	PAM_1	occlusal type II
A0119-001549	PAM_1	occlusal type II
A0119-001551	PAM_1	occlusal type I
A0119-001573	PAM_1	occlusal type I
A0119-001577	PAM_1	occlusal type I
A0119-001578	PAM_1	occlusal type I
A0119-001581	PAM_1	occlusal type I
A0119-001615	PAM_1	occlusal type II
A0119-002337	PAM_1	occlusal type I
A0119-002338	PAM_1	occlusal type II
A0119-002340	PAM_1	occlusal type II
A0119-002385	PAM_1	occlusal type II
A0119-002428	PAM_1	occlusal type I
A0119-002443	PAM_1	occlusal type I
A0119-002447	PAM_1	occlusal type II
A0119-002451	PAM_1	occlusal type II
A0119-002460	PAM_1	occlusal type II
A0119-002513	PAM_1	occlusal type I

Supplemental table 6–Continued

Sample	PAM Clusters	Occlusal type
A0119-002514	PAM_1	occlusal type II
A0119-002515	PAM_1	occlusal type II
A0119-002539	PAM_1	occlusal type II
A0119-002883	PAM_1	occlusal type II
A0119-002885	PAM_1	occlusal type I
A0119-002904	PAM_1	occlusal type II

Supplemental table 7 The annotated KEGG pathways

Ko_ID	Level2	Level3
Ko03010	Translation	Ribosome
Ko00860	Metabolism of cofactors and vitamins	Porphyrin and chlorophyll metabolism
Ko00640	Carbohydrate metabolism	Propanoate metabolism
Ko00540	Glycan biosynthesis and metabolism	Lipopolysaccharide biosynthesis
Ko00030	Carbohydrate metabolism	Pentose phosphate pathway
Ko02020	Signal transduction	Two-component system
Ko00521	Biosynthesis of other secondary metabolites	Streptomycin biosynthesis
Ko00523	Metabolism of terpenoids and polyketides	Polyketide sugar unit biosynthesis
Ko03070	Membrane transport	Bacterial secretion system
Ko00780	Metabolism of cofactors and vitamins	Biotin metabolism
Ko00061	Lipid metabolism	Fatty acid biosynthesis
Ko03440	Replication and repair	Homologous recombination
Ko00520	Carbohydrate metabolism	Amino sugar and nucleotide sugar metabolism
Ko00040	Carbohydrate metabolism	Pentose and glucuronate interconversions
Ko00053	Carbohydrate metabolism	Ascorbate and aldarate metabolism
Ko00900	Metabolism of terpenoids and polyketides	Terpenoid backbone biosynthesis
Ko04146	Transport and catabolism	Peroxisome
Ko00230	Nucleotide metabolism	Purine metabolism
Ko00240	Nucleotide metabolism	Pyrimidine metabolism
Ko00400	Amino acid metabolism	Phenylalanine, tyrosine and tryptophan biosynthesis
Ko00260	Amino acid metabolism	Glycine, serine and threonine metabolism
Ko04112	Cell growth and death	Cell cycle - Caulobacter
Ko03420	Replication and repair	Nucleotide excision repair
Ko00020	Carbohydrate metabolism	Citrate cycle (TCA cycle)
Ko00650	Carbohydrate metabolism	Butanoate metabolism
Ko00720	Energy metabolism	Carbon fixation pathways in prokaryotes
Ko00190	Energy metabolism	Oxidative phosphorylation
Ko02010	Membrane transport	ABC transporters
Ko00270	Amino acid metabolism	Cysteine and methionine metabolism
Ko03060	Folding, sorting and degradation	Protein export
Ko02024	Cellular community - prokaryotes	Quorum sensing
Ko02040	Cell motility	Flagellar assembly
Ko04016	Signal transduction	MAPK signaling pathway - plant
Ko01524	Drug resistance: Antineoplastic	Platinum drug resistance
Ko00250	Amino acid metabolism	Alanine, aspartate and glutamate metabolism
Ko00430	Metabolism of other amino acids	Taurine and hypotaurine metabolism
Ko00910	Energy metabolism	Nitrogen metabolism
Ko00220	Amino acid metabolism	Arginine biosynthesis
Ko04122	Folding, sorting and degradation	Sulfur relay system
Ko00500	Carbohydrate metabolism	Starch and sucrose metabolism
Ko05134	Infectious diseases: Bacterial	Legionellosis

Supplemental table 7–Continued

Ko_ID	Level2	Level3
Ko05132	Infectious diseases: Bacterial	Salmonella infection
Ko04621	Immune system	NOD-like receptor signaling pathway
Ko04626	Environmental adaptation	Plant-pathogen interaction
Ko00970	Translation	Aminoacyl-tRNA biosynthesis
Ko00670	Metabolism of cofactors and vitamins	One carbon pool by folate
Ko04978	Digestive system	Mineral absorption
Ko00760	Metabolism of cofactors and vitamins	Nicotinate and nicotinamide metabolism
Ko00052	Carbohydrate metabolism	Galactose metabolism
Ko03410	Replication and repair	Base excision repair
Ko03430	Replication and repair	Mismatch repair
Ko03030	Replication and repair	DNA replication
Ko05111	Cellular community - prokaryotes	Biofilm formation - Vibrio cholerae
Ko01503	Drug resistance: Antimicrobial	Cationic antimicrobial peptide (CAMP) resistance
Ko01501	Drug resistance: Antimicrobial	beta-Lactam resistance
Ko00740	Metabolism of cofactors and vitamins	Riboflavin metabolism
Ko00770	Metabolism of cofactors and vitamins	Pantothenate and CoA biosynthesis
Ko00710	Energy metabolism	Carbon fixation in photosynthetic organisms
Ko00300	Amino acid metabolism	Lysine biosynthesis
Ko00440	Metabolism of other amino acids	Phosphonate and phosphinate metabolism
Ko00460	Metabolism of other amino acids	Cyanoamino acid metabolism
Ko02060	Membrane transport	Phosphotransferase system (PTS)
Ko00730	Metabolism of cofactors and vitamins	Thiamine metabolism
Ko00195	Energy metabolism	Photosynthesis
Ko00630	Carbohydrate metabolism	Glyoxylate and dicarboxylate metabolism
Ko00790	Metabolism of cofactors and vitamins	Folate biosynthesis
Ko00401	Biosynthesis of other secondary metabolites	Novobiocin biosynthesis
Ko00360	Amino acid metabolism	Phenylalanine metabolism
Ko00960	Biosynthesis of other secondary metabolites	Tropane, piperidine and pyridine alkaloid biosynthesis
Ko00350	Amino acid metabolism	Tyrosine metabolism
Ko00340	Amino acid metabolism	Histidine metabolism
Ko00330	Amino acid metabolism	Arginine and proline metabolism
Ko00550	Glycan biosynthesis and metabolism	Peptidoglycan biosynthesis
Ko02026	Cellular community - prokaryotes	Biofilm formation - Escherichia coli
Ko01051	Metabolism of terpenoids and polyketides	Biosynthesis of ansamycins
Ko00410	Metabolism of other amino acids	beta-Alanine metabolism
Ko00906	Metabolism of terpenoids and polyketides	Carotenoid biosynthesis
Ko00920	Energy metabolism	Sulfur metabolism
Ko05340	Immune diseases	Primary immunodeficiency

Supplemental table 7–Continued

Ko_ID	Level2	Level3
Ko00130	Metabolism of cofactors and vitamins	Ubiquinone and other terpenoid-quinone biosynthesis
Ko01502	Drug resistance: Antimicrobial	Vancomycin resistance
Ko00473	Metabolism of other amino acids	D-Alanine metabolism
Ko01040	Lipid metabolism	Biosynthesis of unsaturated fatty acids
Ko02025	Cellular community - prokaryotes	Biofilm formation - Pseudomonas aeruginosa
Ko00750	Metabolism of cofactors and vitamins	Vitamin B6 metabolism
Ko03018	Folding, sorting and degradation	RNA degradation
Ko05133	Infectious diseases: Bacterial	Pertussis
Ko04217	Cell growth and death	Necroptosis
Ko00311	Biosynthesis of other secondary metabolites	Penicillin and cephalosporin biosynthesis
Ko00620	Carbohydrate metabolism	Pyruvate metabolism
Ko00010	Carbohydrate metabolism	Glycolysis / Gluconeogenesis
Ko00680	Energy metabolism	Methane metabolism
Ko00564	Lipid metabolism	Glycerophospholipid metabolism
Ko00561	Lipid metabolism	Glycerolipid metabolism
Ko00051	Carbohydrate metabolism	Fructose and mannose metabolism
Ko00627	Xenobiotics biodegradation and metabolism	Aminobenzoate degradation
Ko01053	Metabolism of terpenoids and polyketides	Biosynthesis of siderophore group nonribosomal peptides
Ko00562	Carbohydrate metabolism	Inositol phosphate metabolism
Ko04070	Signal transduction	Phosphatidylinositol signaling system
Ko02030	Cell motility	Bacterial chemotaxis
Ko00471	Metabolism of other amino acids	D-Glutamine and D-glutamate metabolism
Ko00480	Metabolism of other amino acids	Glutathione metabolism
Ko00604	Glycan biosynthesis and metabolism	Glycosphingolipid biosynthesis - ganglio series
Ko00511	Glycan biosynthesis and metabolism	Other glycan degradation
Ko00513	Glycan biosynthesis and metabolism	Various types of N-glycan biosynthesis
Ko00531	Glycan biosynthesis and metabolism	Glycosaminoglycan degradation
Ko00603	Glycan biosynthesis and metabolism	Glycosphingolipid biosynthesis - globo and isoglobo series
Ko04142	Transport and catabolism	Lysosome
Ko04213	Aging	Longevity regulating pathway - multiple species
Ko00362	Xenobiotics biodegradation and metabolism	Benzoate degradation
Ko03320	Endocrine system	PPAR signaling pathway
Ko00290	Amino acid metabolism	Valine, leucine and isoleucine biosynthesis
Ko00660	Carbohydrate metabolism	C5-Branched dibasic acid metabolism
Ko05110	Infectious diseases: Bacterial	Vibrio cholerae infection
Ko01523	Drug resistance: Antineoplastic	Antifolate resistance
Ko00310	Amino acid metabolism	Lysine degradation
Ko00380	Amino acid metabolism	Tryptophan metabolism
Ko04212	Aging	Longevity regulating pathway - worm

Supplemental table 7–Continued

Ko_ID	Level2	Level3
Ko01054	Metabolism of terpenoids and polyketides	Nonribosomal peptide structures
Ko04214	Cell growth and death	Apoptosis - fly
Ko00405	Biosynthesis of other secondary metabolites	Phenazine biosynthesis
Ko03013	Translation	RNA transport
Ko04931	Endocrine and metabolic diseases	Insulin resistance
Ko00785	Metabolism of cofactors and vitamins	Lipoic acid metabolism
Ko05418	Cardiovascular diseases	Fluid shear stress and atherosclerosis
Ko00261	Biosynthesis of other secondary metabolites	Monobactam biosynthesis
Ko00524	Biosynthesis of other secondary metabolites	Neomycin, kanamycin and gentamicin biosynthesis
Ko03020	Transcription	RNA polymerase
Ko00450	Metabolism of other amino acids	Selenocompound metabolism
Ko04932	Endocrine and metabolic diseases	Non-alcoholic fatty liver disease (NAFLD)
Ko05012	Neurodegenerative diseases	Parkinson's disease
Ko05010	Neurodegenerative diseases	Alzheimer's disease
Ko04260	Circulatory system	Cardiac muscle contraction
Ko05016	Neurodegenerative diseases	Huntington's disease
Ko05120	Infectious diseases: Bacterial	Epithelial cell signaling in Helicobacter pylori infection
Ko00621	Xenobiotics biodegradation and metabolism	Dioxin degradation
Ko00622	Xenobiotics biodegradation and metabolism	Xylene degradation
Ko05167	Infectious diseases: Viral	Kaposi's sarcoma-associated herpesvirus infection
Ko05168	Infectious diseases: Viral	Herpes simplex infection
Ko05416	Cardiovascular diseases	Viral myocarditis
Ko05161	Infectious diseases: Viral	Hepatitis B
Ko04115	Cell growth and death	p53 signaling pathway
Ko04215	Cell growth and death	Apoptosis - multiple species
Ko05210	Cancers: Specific types	Colorectal cancer
Ko05145	Infectious diseases: Parasitic	Toxoplasmosis
Ko05222	Cancers: Specific types	Small cell lung cancer
Ko05014	Neurodegenerative diseases	Amyotrophic lateral sclerosis (ALS)
Ko05200	Cancers: Overview	Pathways in cancer
Ko05164	Infectious diseases: Viral	Influenza A
Ko04210	Cell growth and death	Apoptosis
Ko05152	Infectious diseases: Bacterial	Tuberculosis
Ko00983	Xenobiotics biodegradation and metabolism	Drug metabolism - other enzymes
Ko03008	Translation	Ribosome biogenesis in eukaryotes
Ko05205	Cancers: Overview	Proteoglycans in cancer
Ko05020	Neurodegenerative diseases	Prion diseases
Ko00071	Lipid metabolism	Fatty acid degradation
Ko00280	Amino acid metabolism	Valine, leucine and isoleucine degradation

Supplemental table 7–Continued

Ko_ID	Level2	Level3
Ko05150	Infectious diseases: Bacterial	Staphylococcus aureus infection
Ko05230	Cancers: Overview	Central carbon metabolism in cancer
Ko04920	Endocrine system	Adipocytokine signaling pathway
Ko04216	Cell growth and death	Ferroptosis
Ko04917	Endocrine system	Prolactin signaling pathway
Ko00908	Metabolism of terpenoids and polyketides	Zeatin biosynthesis
Ko00361	Xenobiotics biodegradation and metabolism	Chlorocyclohexane and chlorobenzene degradation
Ko00625	Xenobiotics biodegradation and metabolism	Chloroalkane and chloroalkene degradation
Ko04910	Endocrine system	Insulin signaling pathway
Ko04922	Endocrine system	Glucagon signaling pathway
Ko04152	Signal transduction	AMPK signaling pathway
Ko04141	Folding, sorting and degradation	Protein processing in endoplasmic reticulum
Ko04918	Endocrine system	Thyroid hormone synthesis
Ko00590	Lipid metabolism	Arachidonic acid metabolism
Ko05203	Cancers: Overview	Viral carcinogenesis
Ko04930	Endocrine and metabolic diseases	Type II diabetes mellitus
Ko05165	Infectious diseases: Viral	Human papillomavirus infection
Ko04072	Signal transduction	Phospholipase D signaling pathway
Ko05231	Cancers: Overview	Choline metabolism in cancer
Ko05146	Infectious diseases: Parasitic	Amoebiasis
Ko04066	Signal transduction	HIF-1 signaling pathway
Ko00525	Biosynthesis of other secondary metabolites	Acarbose and validamycin biosynthesis
Ko00633	Xenobiotics biodegradation and metabolism	Nitrotoluene degradation
Ko05206	Cancers: Overview	MicroRNAs in cancer
Ko00333	Biosynthesis of other secondary metabolites	Prodigiosin biosynthesis
Ko00940	Biosynthesis of other secondary metabolites	Phenylpropanoid biosynthesis
Ko04068	Signal transduction	FoxO signaling pathway
Ko04151	Signal transduction	PI3K-Akt signaling pathway
Ko04964	Excretory system	Proximal tubule bicarbonate reclamation
Ko05204	Cancers: Overview	Chemical carcinogenesis
Ko00982	Xenobiotics biodegradation and metabolism	Drug metabolism - cytochrome P450
Ko00980	Xenobiotics biodegradation and metabolism	Metabolism of xenobiotics by cytochrome P450
Ko05225	Cancers: Specific types	Hepatocellular carcinoma
Ko00965	Biosynthesis of other secondary metabolites	Betalain biosynthesis
Ko00643	Xenobiotics biodegradation and metabolism	Styrene degradation
Ko04211	Aging	Longevity regulating pathway
Ko04011	Signal transduction	MAPK signaling pathway - yeast

Supplemental table 7–Continued

Ko_ID	Level2	Level3
Ko00332	Biosynthesis of other secondary metabolites	Carbapenem biosynthesis
Ko04915	Endocrine system	Estrogen signaling pathway
Ko04657	Immune system	IL-17 signaling pathway
Ko04659	Immune system	Th17 cell differentiation
Ko05215	Cancers: Specific types	Prostate cancer
Ko04612	Immune system	Antigen processing and presentation
Ko04914	Endocrine system	Progesterone-mediated oocyte maturation
Ko00072	Lipid metabolism	Synthesis and degradation of ketone bodies
Ko04013	Signal transduction	MAPK signaling pathway - fly
Ko00966	Biosynthesis of other secondary metabolites	Glucosinolate biosynthesis
Ko00600	Lipid metabolism	Sphingolipid metabolism
Ko00830	Metabolism of cofactors and vitamins	Retinol metabolism
Ko00626	Xenobiotics biodegradation and metabolism	Naphthalene degradation
Ko04727	Nervous system	GABAergic synapse
Ko04724	Nervous system	Glutamatergic synapse
Ko04940	Endocrine and metabolic diseases	Type I diabetes mellitus
Ko00472	Metabolism of other amino acids	D-Arginine and D-ornithine metabolism
Ko05322	Immune diseases	Systemic lupus erythematosus
Ko00121	Lipid metabolism	Secondary bile acid biosynthesis
Ko04974	Digestive system	Protein digestion and absorption
Ko00950	Biosynthesis of other secondary metabolites	Isoquinoline alkaloid biosynthesis
Ko00510	Glycan biosynthesis and metabolism	N-Glycan biosynthesis
Ko01055	Metabolism of terpenoids and polyketides	Biosynthesis of vancomycin group antibiotics
Ko04614	Endocrine system	Renin-angiotensin system
Ko05211	Cancers: Specific types	Renal cell carcinoma
Ko04138	Transport and catabolism	Autophagy - yeast
Ko00591	Lipid metabolism	Linoleic acid metabolism
Ko00565	Lipid metabolism	Ether lipid metabolism
Ko00592	Lipid metabolism	alpha-Linolenic acid metabolism
Ko04113	Cell growth and death	Meiosis - yeast
Ko04973	Digestive system	Carbohydrate digestion and absorption
Ko05219	Cancers: Specific types	Bladder cancer
Ko00140	Lipid metabolism	Steroid hormone biosynthesis
Ko04330	Signal transduction	Notch signaling pathway
Ko04310	Signal transduction	Wnt signaling pathway
Ko05220	Cancers: Specific types	Chronic myeloid leukemia
Ko04340	Signal transduction	Hedgehog signaling pathway
Ko00281	Metabolism of terpenoids and polyketides	Geraniol degradation
Ko05142	Infectious diseases: Parasitic	Chagas disease (American trypanosomiasis)
Ko05143	Infectious diseases: Parasitic	African trypanosomiasis
Ko05100	Infectious diseases: Bacterial	Bacterial invasion of epithelial cells

Supplemental table 7–Continued

Ko_ID	Level2	Level3
Ko03050	Folding, sorting and degradation	Proteasome
Ko04071	Signal transduction	Sphingolipid signaling pathway
Ko04740	Sensory system	Olfactory transduction
Ko00791	Xenobiotics biodegradation and metabolism	Atrazine degradation
Ko00930	Xenobiotics biodegradation and metabolism	Caprolactam degradation
Ko00903	Metabolism of terpenoids and polyketides	Limonene and pinene degradation
Ko00623	Xenobiotics biodegradation and metabolism	Toluene degradation
Ko04728	Nervous system	Dopaminergic synapse
Ko05034	Substance dependence	Alcoholism
Ko05030	Substance dependence	Cocaine addiction
Ko04726	Nervous system	Serotonergic synapse
Ko05031	Substance dependence	Amphetamine addiction
Ko04144	Transport and catabolism	Endocytosis
Ko00642	Xenobiotics biodegradation and metabolism	Ethylbenzene degradation
Ko00515	Glycan biosynthesis and metabolism	Mannose type O-glycan biosynthesis
Ko00944	Biosynthesis of other secondary metabolites	Flavone and flavonol biosynthesis
Ko04976	Digestive system	Bile secretion
Ko00981	Metabolism of terpenoids and polyketides	Insect hormone biosynthesis
Ko00909	Metabolism of terpenoids and polyketides	Sesquiterpenoid and triterpenoid biosynthesis
Ko03022	Transcription	Basal transcription factors
Ko04622	Immune system	RIG-I-like receptor signaling pathway
Ko00120	Lipid metabolism	Primary bile acid biosynthesis
Ko05166	Infectious diseases: Viral	HTLV-I infection
Ko04022	Signal transduction	cGMP-PKG signaling pathway
Ko04218	Cell growth and death	Cellular senescence
Ko04020	Signal transduction	Calcium signaling pathway
Ko01052	Metabolism of terpenoids and polyketides	Type I polyketide structures
Ko04721	Nervous system	Synaptic vesicle cycle

Supplemental table 8 Important metabolites identified by OPLS-DA

Metabolites	P value	Vip score	Annotation	Enriched group
kynurenate	0.029865432	1.663686432	Amino Acid	OT II
gluconate	0.017649312	1.37896551	Xenobiotics	OT II
adenosine	0.038721723	-1.889011064	Nucleotide	OT I
uracil	0.010300896	-2.130233461	Nucleotide	OT I
fructose	0.008351723	1.728015183	Carbohydrate	OT II
pipecolate	0.007426722	1.48172048	Amino Acid	OT II
N-acetylalanine	0.030198738	2.087144659	Amino Acid	OT II
urate	0.023644053	1.582906452	Nucleotide	OT II
N1-methyladenosine	0.027447136	1.37769156	Nucleotide	OT II
azelate (C9-DC)	0.015326691	2.100457029	Lipid	OT II
phenylacetate	0.037533948	-1.735092685	Amino Acid	OT I
suberate (C8-DC)	0.016454947	2.05690146	Lipid	OT II
4-guanidinobutanoate	0.000589294	2.200176188	Amino Acid	OT II
creatine phosphate	0.005119105	1.757172059	Amino Acid	OT II
imidazole lactate	0.013788472	1.418442061	Amino Acid	OT II
2-isopropylmalate	0.028821314	1.863792449	Xenobiotics	OT II
1-stearoyl-2-arachidonoyl-GPI (18:0/20:4)	0.035018104	1.980193576	Lipid	OT II
mevalonolactone	0.001897874	2.041487334	Lipid	OT II
adipate (C6-DC)	0.014233303	2.069912472	Lipid	OT II
triethanolamine	0.00892237	1.944900883	Xenobiotics	OT II
3-hydroxydecanoate	0.000958901	1.860490716	Lipid	OT II
galactonate	0.047839569	1.469131044	Carbohydrate	OT II
dodecanedioate (C12-DC)	0.025012094	1.726923843	Lipid	OT II
2-hydroxyadipate	0.0182763	1.742714851	Lipid	OT II
4-imidazoleacetate	0.017848315	1.628489098	Amino Acid	OT II
N-acetylisoleucine	0.028580862	1.949188692	Amino Acid	OT II
N6-carbamoylthreonyladenosine	0.043499533	1.24822325	Nucleotide	OT II
orotidine	0.042619476	1.326055895	Nucleotide	OT II
phenylacetylglutamine	0.006442985	1.871693377	Peptide	OT II
2-methylbutyrylcarnitine (C5)	0.00196522	1.59244896	Amino Acid	OT II
undecanedioate (C11-DC)	0.022761812	1.955984932	Lipid	OT II
2S,3R-dihydroxybutyrate	0.008677334	1.434034545	Lipid	OT II
sulfate	0.016736342	1.468524782	Xenobiotics	OT II
histidylalanine	0.037989764	-2.464171051	Peptide	OT I
phenylalanylglycine	0.026096545	-2.075067873	Peptide	OT I
tyrosylglycine	0.041619308	-1.959259059	Peptide	OT I
arabonate/xylonate	0.036016092	1.115719486	Carbohydrate	OT II

Supplemental table 8–Continued

**Metabolites**	**P value**	**Vip score**	**Annotation**	**Enriched group**
C-glycosyltryptophan	0.023651218	1.538604589	Amino Acid	OT II
arabitol/xylitol	0.012071482	1.985615231	Carbohydrate	OT II
lyxonate	0.014659831	1.93339963	Carbohydrate	OT II
dodecenedioate (C12:1-DC)	0.01529448	2.361597122	Lipid	OT II
N-Acetylisoputreanine	0.029342525	1.50055444	Amino Acid	OT II
vitamin D3 sulfate	0.021004322	1.898553774	Cofactors and Vitamins	OT II
hydroxy-N6,N6,N6-trimethyllysine	0.022638259	1.823943909	Amino Acid	OT II

Supplemental table 9 Significant linkages between microbes and metabolites via functional pathways

Types	Mediation.Pval	Index
Indirect (ab)210	0.019706109	Prevotella_timonensis~Ko01503~phenylacetate
Direct (c')210	0.046612636	Prevotella_timonensis~Ko01503~phenylacetate
Total (c)210	0.00060511	Prevotella_timonensis~Ko01503~phenylacetate
Indirect (ab)214	0.02464297	Prevotella_timonensis~Ko04070~phenylacetate
Direct (c')214	0.03299212	Prevotella_timonensis~Ko04070~phenylacetate
Total (c)214	0.00060511	Prevotella_timonensis~Ko04070~phenylacetate
Indirect (ab)216	0.029539957	Streptococcus_cristatus~Ko00071~phenylacetate
Direct (c')216	6.7578E-05	Streptococcus_cristatus~Ko00071~phenylacetate
Total (c)216	6.30871E-07	Streptococcus_cristatus~Ko00071~phenylacetate
Indirect (ab)217	0.028715349	Streptococcus_cristatus~Ko00350~phenylacetate
Indirect (ab)218	0.014514428	Streptococcus_cristatus~Ko00480~phenylacetate
Direct (c')218	2.93657E-05	Streptococcus_cristatus~Ko00480~phenylacetate
Total (c)218	6.30871E-07	Streptococcus_cristatus~Ko00480~phenylacetate
Indirect (ab)219	0.027036163	Streptococcus_cristatus~Ko00564~phenylacetate
Direct (c')219	4.90092E-05	Streptococcus_cristatus~Ko00564~phenylacetate
Total (c)219	6.30871E-07	Streptococcus_cristatus~Ko00564~phenylacetate
Indirect (ab)220	0.025913268	Streptococcus_cristatus~Ko00920~phenylacetate
Direct (c')220	3.86799E-05	Streptococcus_cristatus~Ko00920~phenylacetate
Total (c)220	6.30871E-07	Streptococcus_cristatus~Ko00920~phenylacetate
Indirect (ab)221	0.045861042	Streptococcus_cristatus~Ko01501~phenylacetate
Direct (c')221	3.03897E-05	Streptococcus_cristatus~Ko01501~phenylacetate
Total (c)221	6.30871E-07	Streptococcus_cristatus~Ko01501~phenylacetate
Indirect (ab)222	0.015401195	Streptococcus_cristatus~Ko01503~phenylacetate
Direct (c')222	0.000174035	Streptococcus_cristatus~Ko01503~phenylacetate
Total (c)222	6.30871E-07	Streptococcus_cristatus~Ko01503~phenylacetate
Indirect (ab)223	0.005622579	Streptococcus_cristatus~Ko02010~phenylacetate
Direct (c')223	0.00245609	Streptococcus_cristatus~Ko02010~phenylacetate
Total (c)223	6.30871E-07	Streptococcus_cristatus~Ko02010~phenylacetate
Indirect (ab)224	0.006910464	Streptococcus_cristatus~Ko02020~phenylacetate
Direct (c')224	0.020956689	Streptococcus_cristatus~Ko02020~phenylacetate
Total (c)224	6.30871E-07	Streptococcus_cristatus~Ko02020~phenylacetate
Indirect (ab)225	0.025517717	Streptococcus_cristatus~Ko02026~phenylacetate
Direct (c')225	0.000598289	Streptococcus_cristatus~Ko02026~phenylacetate
Total (c)225	6.30871E-07	Streptococcus_cristatus~Ko02026~phenylacetate
Indirect (ab)226	0.040739577	Streptococcus_cristatus~Ko04070~phenylacetate
Direct (c')226	1.47658E-05	Streptococcus_cristatus~Ko04070~phenylacetate
Total (c)226	6.30871E-07	Streptococcus_cristatus~Ko04070~phenylacetate
Indirect (ab)227	0.01780521	Streptococcus_cristatus~Ko04122~phenylacetate
Direct (c')227	6.79617E-05	Streptococcus_cristatus~Ko04122~phenylacetate
Total (c)227	6.30871E-07	Streptococcus_cristatus~Ko04122~phenylacetate
Indirect (ab)386	0.046302018	Veillonella_sp._HPA0037~Ko03010~triethanolamine
Direct (c')386	0.039251128	Veillonella_sp._HPA0037~Ko03010~triethanolamine
Total (c)386	0.000162695	Veillonella_sp._HPA0037~Ko03010~triethanolamine
Indirect (ab)638	0.036428912	Veillonella_sp._ICM51a~Ko03010~phenylalanylglycine
Direct (c')638	0.003675476	Veillonella_sp._ICM51a~Ko03010~phenylalanylglycine

Supplemental table 9–Continued

Types	Mediation.Pval	Index
Total (c)638	0.000398124	Veillonella_sp._ICM51a~Ko03010~phenylalanylglycine
Indirect (ab)698	0.017866447	Prevotella_pallens~Ko03010~phenylalanylglycine
Direct (c')698	0.005515445	Prevotella_pallens~Ko03010~phenylalanylglycine
Total (c)698	0.000619552	Prevotella_pallens~Ko03010~phenylalanylglycine
Indirect (ab)713	0.021848867	Prevotella_disiens~Ko03010~phenylalanylglycine
Direct (c')713	0.00037132	Prevotella_disiens~Ko03010~phenylalanylglycine
Total (c)713	3.242E-07	Prevotella_disiens~Ko03010~phenylalanylglycine
Indirect (ab)753	0.033197229	Haemophilus_parahaemolyticus~Ko01501~phenylalanylglycine
Direct (c')753	0.008130571	Haemophilus_parahaemolyticus~Ko01501~phenylalanylglycine
Total (c)753	0.002914082	Haemophilus_parahaemolyticus~Ko01501~phenylalanylglycine
Indirect (ab)761	0.048141085	Haemophilus_parahaemolyticus~Ko04122~phenylalanylglycine
Direct (c')761	0.040721021	Haemophilus_parahaemolyticus~Ko04122~phenylalanylglycine
Total (c)761	0.002914082	Haemophilus_parahaemolyticus~Ko04122~phenylalanylglycine
Indirect (ab)828	0.020719878	Haemophilus_influenzae~Ko01501~phenylalanylglycine
Direct (c')828	0.002657381	Haemophilus_influenzae~Ko01501~phenylalanylglycine
Total (c)828	3.94598E-09	Haemophilus_influenzae~Ko01501~phenylalanylglycine
Indirect (ab)831	0.001582536	Haemophilus_influenzae~Ko02020~phenylalanylglycine
Direct (c')831	0.029069446	Haemophilus_influenzae~Ko02020~phenylalanylglycine
Total (c)831	3.94598E-09	Haemophilus_influenzae~Ko02020~phenylalanylglycine
Indirect (ab)860	0.008241448	Neisseria_sp._HMSC03D10~Ko02010~phenylalanylglycine
Direct (c')860	0.036359369	Neisseria_sp._HMSC03D10~Ko02010~phenylalanylglycine
Total (c)860	0.000196524	Neisseria_sp._HMSC03D10~Ko02010~phenylalanylglycine
Indirect (ab)861	0.001213104	Neisseria_sp._HMSC03D10~Ko02020~phenylalanylglycine
Direct (c')861	0.049351287	Neisseria_sp._HMSC03D10~Ko02020~phenylalanylglycine
Total (c)861	0.000196524	Neisseria_sp._HMSC03D10~Ko02020~phenylalanylglycine
Indirect (ab)862	0.015256409	Neisseria_sp._HMSC03D10~Ko02026~phenylalanylglycine
Direct (c')862	0.010523396	Neisseria_sp._HMSC03D10~Ko02026~phenylalanylglycine
Total (c)862	0.000196524	Neisseria_sp._HMSC03D10~Ko02026~phenylalanylglycine
Indirect (ab)863	0.040876411	Neisseria_sp._HMSC03D10~Ko03010~phenylalanylglycine
Direct (c')863	0.005783571	Neisseria_sp._HMSC03D10~Ko03010~phenylalanylglycine
Total (c)863	0.000196524	Neisseria_sp._HMSC03D10~Ko03010~phenylalanylglycine
Indirect (ab)866	0.015965679	Neisseria_sp._HMSC03D10~Ko04122~phenylalanylglycine
Direct (c')866	0.007015961	Neisseria_sp._HMSC03D10~Ko04122~phenylalanylglycine
Total (c)866	0.000196524	Neisseria_sp._HMSC03D10~Ko04122~phenylalanylglycine
Indirect (ab)882	0.032956086	Streptococcus_cristatus~Ko00350~phenylalanylglycine
Direct (c')882	0.014977102	Streptococcus_cristatus~Ko00350~phenylalanylglycine
Total (c)882	0.00188907	Streptococcus_cristatus~Ko00350~phenylalanylglycine
Indirect (ab)883	0.026467234	Streptococcus_cristatus~Ko00480~phenylalanylglycine
Direct (c')883	0.016720808	Streptococcus_cristatus~Ko00480~phenylalanylglycine
Total (c)883	0.00188907	Streptococcus_cristatus~Ko00480~phenylalanylglycine
Indirect (ab)884	0.035011401	Streptococcus_cristatus~Ko00564~phenylalanylglycine
Direct (c')884	0.01578242	Streptococcus_cristatus~Ko00564~phenylalanylglycine
Total (c)884	0.00188907	Streptococcus_cristatus~Ko00564~phenylalanylglycine
Indirect (ab)886	0.034332517	Streptococcus_cristatus~Ko00920~phenylalanylglycine
Direct (c')886	0.015713976	Streptococcus_cristatus~Ko00920~phenylalanylglycine
Total (c)886	0.00188907	Streptococcus_cristatus~Ko00920~phenylalanylglycine

Supplemental table 9–Continued

**Types**	**Mediation.Pval**	**Index**
Indirect (ab)888	0.013527698	Streptococcus_cristatus~Ko01501~phenylalanylglycine
Direct (c')888	0.02714093	Streptococcus_cristatus~Ko01501~phenylalanylglycine
Total (c)888	0.00188907	Streptococcus_cristatus~Ko01501~phenylalanylglycine
Indirect (ab)889	0.012754485	Streptococcus_cristatus~Ko01503~phenylalanylglycine
Direct (c')889	0.030084148	Streptococcus_cristatus~Ko01503~phenylalanylglycine
Total (c)889	0.00188907	Streptococcus_cristatus~Ko01503~phenylalanylglycine
Indirect (ab)892	0.019409092	Streptococcus_cristatus~Ko02026~phenylalanylglycine
Direct (c')892	0.039239673	Streptococcus_cristatus~Ko02026~phenylalanylglycine
Total (c)892	0.00188907	Streptococcus_cristatus~Ko02026~phenylalanylglycine
Indirect (ab)893	0.037056632	Streptococcus_cristatus~Ko03010~phenylalanylglycine
Direct (c')893	0.013139642	Streptococcus_cristatus~Ko03010~phenylalanylglycine
Total (c)893	0.00188907	Streptococcus_cristatus~Ko03010~phenylalanylglycine
Indirect (ab)896	0.042475053	Streptococcus_cristatus~Ko04122~phenylalanylglycine
Direct (c')896	0.012703624	Streptococcus_cristatus~Ko04122~phenylalanylglycine
Total (c)896	0.00188907	Streptococcus_cristatus~Ko04122~phenylalanylglycine
Indirect (ab)923	0.026930626	Veillonella_sp._ICM51a~Ko03010~tyrosylglycine
Direct (c')923	0.018116218	Veillonella_sp._ICM51a~Ko03010~tyrosylglycine
Total (c)923	0.00201315	Veillonella_sp._ICM51a~Ko03010~tyrosylglycine
Indirect (ab)951	0.014519887	Prevotella_pallens~Ko03010~tyrosylglycine
Direct (c')951	0.020499468	Prevotella_pallens~Ko03010~tyrosylglycine
Total (c)951	0.002401192	Prevotella_pallens~Ko03010~tyrosylglycine
Indirect (ab)965	0.002664771	Prevotella_disiens~Ko03010~tyrosylglycine
Direct (c')965	0.013134226	Prevotella_disiens~Ko03010~tyrosylglycine
Total (c)965	9.1767E-05	Prevotella_disiens~Ko03010~tyrosylglycine
Indirect (ab)988	0.049619007	Haemophilus_parahaemolyticus~Ko01501~tyrosylglycine
Direct (c')988	0.001441668	Haemophilus_parahaemolyticus~Ko01501~tyrosylglycine
Total (c)988	0.001007656	Haemophilus_parahaemolyticus~Ko01501~tyrosylglycine
Indirect (ab)1056	0.03999667	Haemophilus_influenzae~Ko00920~tyrosylglycine
Direct (c')1056	0.000230142	Haemophilus_influenzae~Ko00920~tyrosylglycine
Total (c)1056	4.13603E-06	Haemophilus_influenzae~Ko00920~tyrosylglycine
Indirect (ab)1058	0.01252057	Haemophilus_influenzae~Ko01501~tyrosylglycine
Direct (c')1058	0.009736052	Haemophilus_influenzae~Ko01501~tyrosylglycine
Total (c)1058	4.13603E-06	Haemophilus_influenzae~Ko01501~tyrosylglycine
Indirect (ab)1072	0.013188764	Neisseria_sp._HMSC03D10~Ko01501~tyrosylglycine
Direct (c')1072	0.036543773	Neisseria_sp._HMSC03D10~Ko01501~tyrosylglycine
Total (c)1072	0.000312631	Neisseria_sp._HMSC03D10~Ko01501~tyrosylglycine
Indirect (ab)1073	0.011486827	Neisseria_sp._HMSC03D10~Ko01503~tyrosylglycine
Direct (c')1073	0.028194932	Neisseria_sp._HMSC03D10~Ko01503~tyrosylglycine
Total (c)1073	0.000312631	Neisseria_sp._HMSC03D10~Ko01503~tyrosylglycine
Indirect (ab)1074	0.015000535	Neisseria_sp._HMSC03D10~Ko02010~tyrosylglycine
Direct (c')1074	0.026979461	Neisseria_sp._HMSC03D10~Ko02010~tyrosylglycine
Total (c)1074	0.000312631	Neisseria_sp._HMSC03D10~Ko02010~tyrosylglycine
Indirect (ab)1075	0.00218066	Neisseria_sp._HMSC03D10~Ko02020~tyrosylglycine
Direct (c')1075	0.047300143	Neisseria_sp._HMSC03D10~Ko02020~tyrosylglycine
Total (c)1075	0.000312631	Neisseria_sp._HMSC03D10~Ko02020~tyrosylglycine
Indirect (ab)1076	0.020327465	Neisseria_sp._HMSC03D10~Ko02026~tyrosylglycine

Supplemental table 9–Continued

Types	Mediation.Pval	Index
Direct (c')1076	0.006601924	Neisseria_sp._HMSC03D10~Ko02026~tyrosylglycine
Total (c)1076	0.000312631	Neisseria_sp._HMSC03D10~Ko02026~tyrosylglycine
Indirect (ab)1077	0.022507713	Neisseria_sp._HMSC03D10~Ko03010~tyrosylglycine
Direct (c')1077	0.007240313	Neisseria_sp._HMSC03D10~Ko03010~tyrosylglycine
Total (c)1077	0.000312631	Neisseria_sp._HMSC03D10~Ko03010~tyrosylglycine
Indirect (ab)1079	0.035146144	Neisseria_sp._HMSC03D10~Ko04122~tyrosylglycine
Direct (c')1079	0.00604056	Neisseria_sp._HMSC03D10~Ko04122~tyrosylglycine
Total (c)1079	0.000312631	Neisseria_sp._HMSC03D10~Ko04122~tyrosylglycine
Indirect (ab)1100	0.019783516	Streptococcus_cristatus~Ko01501~tyrosylglycine
Direct (c')1100	0.019491872	Streptococcus_cristatus~Ko01501~tyrosylglycine
Total (c)1100	0.001308417	Streptococcus_cristatus~Ko01501~tyrosylglycine
Indirect (ab)1101	0.017369504	Streptococcus_cristatus~Ko01503~tyrosylglycine
Direct (c')1101	0.022500241	Streptococcus_cristatus~Ko01503~tyrosylglycine
Total (c)1101	0.001308417	Streptococcus_cristatus~Ko01503~tyrosylglycine
Indirect (ab)1104	0.027878092	Streptococcus_cristatus~Ko02026~tyrosylglycine
Direct (c')1104	0.027626829	Streptococcus_cristatus~Ko02026~tyrosylglycine
Total (c)1104	0.001308417	Streptococcus_cristatus~Ko02026~tyrosylglycine
Indirect (ab)1105	0.034674116	Streptococcus_cristatus~Ko03010~tyrosylglycine
Direct (c')1105	0.012902355	Streptococcus_cristatus~Ko03010~tyrosylglycine
Total (c)1105	0.001308417	Streptococcus_cristatus~Ko03010~tyrosylglycine
Indirect (ab)1106	0.035830901	Streptococcus_cristatus~Ko04112~tyrosylglycine
Direct (c')1106	0.038031081	Streptococcus_cristatus~Ko04112~tyrosylglycine
Total (c)1106	0.001308417	Streptococcus_cristatus~Ko04112~tyrosylglycine
Indirect (ab)1136	0.048004612	Veillonella_sp._ICM51a~Ko02010~uracil
Direct (c')1136	0.038770229	Veillonella_sp._ICM51a~Ko02010~uracil
Total (c)1136	5.75582E-05	Veillonella_sp._ICM51a~Ko02010~uracil
Indirect (ab)1138	0.032230805	Veillonella_sp._ICM51a~Ko02026~uracil
Direct (c')1138	0.041431344	Veillonella_sp._ICM51a~Ko02026~uracil
Total (c)1138	5.75582E-05	Veillonella_sp._ICM51a~Ko02026~uracil
Indirect (ab)1193	0.037198674	Veillonella_atypica~Ko02020~uracil
Direct (c')1193	0.047367984	Veillonella_atypica~Ko02020~uracil
Total (c)1193	4.14213E-05	Veillonella_atypica~Ko02020~uracil
Indirect (ab)1201	0.005934684	Haemophilus_influenzae~Ko02020~uracil
Direct (c')1201	0.004213493	Haemophilus_influenzae~Ko02020~uracil
Total (c)1201	1.42389E-06	Haemophilus_influenzae~Ko02020~uracil
Indirect (ab)1234	0.044748672	Neisseria_sp._HMSC03D10~Ko02026~uracil
Direct (c')1234	0.035502413	Neisseria_sp._HMSC03D10~Ko02026~uracil
Total (c)1234	0.000225565	Neisseria_sp._HMSC03D10~Ko02026~uracil
Indirect (ab)1251	0.047054288	Selenomonas_sp._CM52~Ko00480~arabonate
Direct (c')1251	0.025752	Selenomonas_sp._CM52~Ko00480~arabonate
Total (c)1251	0.000524676	Selenomonas_sp._CM52~Ko00480~arabonate
Indirect (ab)1252	0.040882372	Selenomonas_sp._CM52~Ko00564~arabonate
Direct (c')1252	0.033882389	Selenomonas_sp._CM52~Ko00564~arabonate
Total (c)1252	0.000524676	Selenomonas_sp._CM52~Ko00564~arabonate
Indirect (ab)1415	0.008009918	Prevotella_disiens~Ko03010~lyxonate
Direct (c')1415	0.039859907	Prevotella_disiens~Ko03010~lyxonate

Supplemental table 9–Continued

Types	Mediation.Pval	Index
Total (c)1415	0.001198116	Prevotella_disiens~Ko03010~lyxonate
Indirect (ab)1416	0.043031005	Prevotella_disiens~Ko04112~lyxonate
Direct (c')1416	0.026390313	Prevotella_disiens~Ko04112~lyxonate
Total (c)1416	0.001198116	Prevotella_disiens~Ko04112~lyxonate
Indirect (ab)1433	0.044363274	Streptococcus_cristatus~Ko03010~lyxonate
Direct (c')1433	0.022227571	Streptococcus_cristatus~Ko03010~lyxonate
Total (c)1433	0.003821808	Streptococcus_cristatus~Ko03010~lyxonate
Indirect (ab)1434	0.009412453	Streptococcus_cristatus~Ko04112~lyxonate
Direct (c')1434	0.0492413	Streptococcus_cristatus~Ko04112~lyxonate
Total (c)1434	0.003821808	Streptococcus_cristatus~Ko04112~lyxonate
